# The Effects of Harvesting Media on Biological Characteristics and Repair Potential of Neural Stem Cells after Traumatic Brain Injury

**DOI:** 10.1371/journal.pone.0107865

**Published:** 2014-09-23

**Authors:** Shengliang Liu, Zhuying Li, Jin Fu, Liang Sun, Fengyan Xu, Toshihide Harada, Yu Lou, Ming Chu, Qi Sun, Kun Xu, Rui Zhang, Lianhong Jin, Hui Xiao, Shuliang Wu

**Affiliations:** 1 Department of Anatomy, School of Basic Medical Sciences, Harbin Medical University, Harbin, Heilongjiang Province, China; 2 The First Affiliated Hospital of Heilongjiang University of Chinese Medicine, Harbin, Heilongjiang Province, China; 3 The Second Affiliated Hospital of Harbin Medical University, Harbin, Heilongjiang Province, China; 4 Prefectural University of Hiroshima, Hiroshima, Japan; 5 The First Affiliated Hospital of Harbin Medical University, Harbin, Heilongjiang Province, China; 6 The Second Affiliated Hospital of Heilongjiang University of Chinese Medicine, Harbin, Heilongjiang Province, China; Temple University School of Medicine, United States of America

## Abstract

Various solutions are utilized widely for the isolation, harvesting, sorting, testing and transplantation of neural stem cells (NSCs), whereas the effects of harvesting media on the biological characteristics and repair potential of NSCs remain unclear. To examine some of these effects, NSCs were isolated from cortex of E14.5 mice and exposed to the conventional harvesting media [0.9% saline (Saline), phosphate-buffered saline (PBS) or artificial cerebrospinal fluid (ACSF)] or the proliferation culture medium (PCM) for different durations at 4°C. Treated NSCs were grafted by in situ injection into the lesion sites of traumatic brain injury (TBI) mice. In vitro, harvesting media-exposed NSCs displayed time-dependent reduction of viability and proliferation. S phase entry decreased in harvesting media-exposed cells, which was associated with upregulation of p53 protein and downregulation of cyclin E1 protein. Moreover, harvesting media exposure induced the necrosis and apoptosis of NSCs. The levels of Fas-L, cleaved caspase 3 and 8 were increased, which suggests that the death receptor signaling pathway is involved in the apoptosis of NSCs. In addition, exposure to Saline did not facilitate the neuronal differentiation of NSCs, suggesting that Saline exposure may be disadvantageous for neurogenesis. In vivo, NSC-mediated functional recovery in harvesting media-exposed NSC groups was notably attenuated in comparison with the PCM-exposed NSC group. In conclusion, harvesting media exposure modulates the biological characteristics and repair potential of NSCs after TBI. Our results suggest that insight of the effects of harvesting media exposure on NSCs is critical for developing strategies to assure the successful long-term engraftment of NSCs.

## Introduction

Traumatic brain injury (TBI) remains a major cause of morbidity, mortality and long-term disability in children and young adults [1], [2]. It imposes a significant threat to the lives of patients, remains a profound and long-lasting social and economic consequence and is poorly treated by currently available drugs [2], [3]. Neural stem cell (NSC) transplantation provides an attractive alternative option for treating this condition. Transplanted NSCs have the capacity to migrate long distances to lesion sites and to improve functional recovery after brain injury. Under appropriate conditions, they can differentiate into neuronal and glial lineages and induce the regeneration of damaged brain tissue [4], [5]. Although NSCs have shown promise for cell replacement in brain injury, NSC replacement therapies face many obstacles, including low cell viability, lack of control of stem-cell fate and low levels of cell engraftment after transplantation [5]–[7] These difficulties might result partly from the poor quality of NSCs in vitro and ultimately lead to low levels of cell engraftment. Successful NSC grafting requires, above all, that NSCs need be able to survive and proliferate and that their therapeutic progeny function well [8], [9]. From extraction to transplantation, NSCs experience various human interventions, such as isolation, collection, testing, processing, preservation, storage and distribution in different solutions for different durations. In previous studies, some widely used solutions, including 0.9% saline (Saline), 0.01 M phosphate buffered saline (PBS) and artificial cerebrospinal fluid (ACSF), were employed as the harvesting media for NSC transplantation [10]–[16]. However, the possible effects of the harvesting media exposure on NSCs have not been addressed. In the current study, aiming to optimize the NSC transplantation regimen, maximize the NSC therapeutic potential and develop strategies to assure successful long-term engraftment of NSCs, we investigated the effects of harvesting media exposure on the biological properties and repair function of NSCs. We found that exposure to harvesting media modulated the viability and proliferation of NSCs in a time dependent manner and consequently attenuated the repair potential of NSCs for TBI.

## Materials and Methods

### Ethics Statement

All animal procedures were performed in strict accordance with the guidelines established by the Harbin Medical University Animal Care and Use Committee and approved by the Harbin Medical University Animal Care and Use Committee.

### Preparation of NSCs

Primary NSCs were isolated from 14.5-day-old embryos (E14.5) of C57BL/6 mice for *in vitro* studies and from enhanced green fluorescence protein (EGFP)-transgenic mice [C57BL/6-Tg (CAG-EGFP) 1Osb/J] for *in vivo* studies (all from the Institute of Model Animal, Nan Jing, China). For proliferation, dissociated single cells were cultured in the proliferation culture medium (PCM) containing Dulbecco's modified Eagle's medium/F-12 (DMEM/F-12) (Invitrogen, Carlsbad, CA, USA) supplemented with 1% B27 (Invitrogen), 20 ng/ml basic fibroblast growth factor (bFGF) (Sigma, St. Louis, MO, USA), 20 ng/ml epidermal growth factor (EGF) (Sigma) and 100 U/ml penicillin/streptomycin (Invitrogen). Primary neurospheres were formed after 5–7 days of culture. NSCs at passages 3–5 were used in the present study. For differentiation, NSCs were seeded onto poly-D-lysine (150 µg/ml, Sigma) and laminin (20 µg/ml, Invitrogen) -coated coverslips, cultured in the differentiation medium for 7 to 14 days and analyzed by immunocytochemistry. The differentiation medium contains Neurobasal medium (Invitrogen) supplemented with 1% B27 supplement, 10 ng/ml bFGF, 100 µM dibutyryl cyclic-adenosine monophosphate (dBcAMP) (Sigma) and 100 U/ml penicillin/streptomycin (Invitrogen), which is an efficient neuronal differentiation medium [17], [18].

### Experimental design

In vitro, cells were exposed to one of the various harvesting media [0.9% saline (Saline), 0.01 M PBS (137 mM NaCl, 2.7 mM KCl, 10 mM Na_2_HPO_4_, 2 mM KH_2_PO_4_) or ACSF (119 mM NaCl, 26.2 mM NaHCO_3_, 2.5 mM KCl, 1 mM NaH_2_PO_4_, 1.3 mM MgCl_2_, 10 mM glucose)] or the PCM for 0, 1, 2, 4, 6, 8, 12, 16 or 24 h at 4°C. The pH of the harvesting media and the PCM is 7.2–7.4 and osmolarity is 280-310 mOsm, respectively. The viability, proliferation, differentiation, cell cycle progression, apoptosis and necrosis of cells were monitored by appropriate methods. Exposed to the various harvesting media or the PCM for 6 h, NSCs derived from EGFP-transgenic mice were grafted into lesion sites by in situ injection on day 7 post-TBI. The repair potential of treated NSCs was assessed by testing the morphological and functional recovery of mice.

### Cell viability and proliferation assay

Neurospheres were dissociated into single cells using 0.2% trypsin (Invitrogen) and exposed to the harvesting media or the PCM according to the protocol described above. The trypan blue exclusion assay was used to determine the viability of cells in each group. Briefly, treated cell suspensions were thoroughly mixed with equal volumes of 0.4% trypan blue solution (Gibco, Grand Island, NY, USA) and incubated for 3 min at 37°C. The numbers of surviving (non-stained) and dead (stained) cells were estimated using a hemocytometer chamber. The proliferation of harvesting media-exposed cells was assessed by the BrdU incorporation assay. Dissociated cells or neurospheres were plated on poly-lysine/laminin coated coverslips and incubated in proliferation medium containing 10 µM BrdU (Invitrogen) for 3 h at 37°C prior to immunocytochemical analysis.

### Flow cytometry analysis

Cell cycle analysis was performed as described previously [19] with slight modification. Briefly, treated cells were fixed with ice-cold 70% (w/v) ethanol at 4°C for 2 h, washed with cold PBS and re-suspended in staining buffer containing 40 µg/ml propidium iodide (PI) and 100 µg/ml RNase A (all from BD Biosciences, San Jose, CA, USA) in PBS (pH 7.4). After incubating for 30 min in the dark at room temperature, the cells were analyzed by flow cytometry (BD Biosciences). The percentages of cells in the G_1_, S, G_2_ and M phases of the cell cycle were determined using ModFit LT 3.0 (BD Biosciences).

The Annexin V-fluorescein isothiocyanate (FITC) binding assay was performed according to the manufacturer's instructions using the Annexin V-FITC/PI detection kit (BD Biosciences). Briefly, treated cells were centrifugated and re-suspended in 100 µl of binding buffer at a density of 1×10^6^ cells per ml and incubated with 5 µl of FITC-conjugated Annexin-V and 5 µl of PI for 30 min at room temperature in the dark. Four hundred microliters of 1× binding buffer was added to each sample tube, and the samples were immediately analyzed and classified as surviving (Annexin^−^/PI^−^), early-apoptotic (Annexin^+^/PI^−^), late-apoptotic (Annexin^+^/PI^+^) or necrotic cells (PI^+^) by flow cytometry (BD Biosciences).

### TUNEL assay

The terminal deoxynucleotidyl transferase (TdT) dilutions of cell-delivered dUTP nick 3′-end DNA labeling (TUNEL) assay was performed according to the manufacturer's instructions (Roche Molecular Biochemicals, Germany). The TUNEL-positive and hematoxylin-labeled cells were counted in 10 random fields (×100). The apoptosis rate was calculated as the percentage of TUNEL-positive cells.

### Transmission electron microscopy (TEM)

Treated cells and damaged brain tissue from TBI mice were fixed with 2.5% glutaraldehyde in 0.1 M PBS (pH 7.4) for 8 h at 4°C and further fixed with 1% osmium tetroxide in 0.1 M PBS (pH 7.4) for 8 h at 4°C. The cells and tissues were gradually dehydrated in increasing concentrations of acetone and embedded in Epon812. Ultrathin sections (60 nm) were cut on a Leica Ultra-CUT (Ultra-Microtome, Leica Microsystems GmbH, Wetzlar, Germany) and were electron-stained with uranyl acetate and lead citrate. Sections were examined with TEM (H-7650; Hitachi, Osaka, Japan). The images were captured with a MegaView III CCD camera (Soft Imaging System, Lakewood, CO, USA).

### Mitochondrial Membrane Potential (MMP)

Dissociated NSCs were plated at 1×10^4^ cells/ml on poly-lysine/laminin coated coverslips for 24 h and further exposed to the harvesting media (Saline, PBS or ACSF) for 12 h. As positive control, adherent NSCs were treated with 5 µM staurosporine (Sigma) for 12 h. Rhodamine 123 (Excitation 504/Emission 534) labeling solution (Invitrogen; 10 µg/ml) was added to live cells and incubated for 1 hour. Stained cells were visualized and images were captured using confocal microscopy (IX83, Olympus, Japan).

### Immunocytochemical staining

Immunocytochemical analyses were performed on cells as described [20]. After washing with PBS, the samples were fixed in 4% paraformaldehyde for 20 min, permeabilized in 0.5% Triton X-100 for 15 min and blocked with 3% bovine serum albumin (BSA, Invitrogen) in PBS for 20 min. The samples were incubated with anti-nestin or anti-β-tubulin-III primary antibody (both rabbit IgG; 1∶200; Chemicon, El Segundo, CA, USA) overnight at 4°C. After washing with wash buffer 0.1% BSA in PBS, the samples were incubated with secondary antibody (goat anti rabbit IgG-Alexa Fluor 488 or goat anti rabbit IgG-Alexa Fluor 594; 1∶5000; Molecular Probes, Invitrogen) for 20 min at room temperature in the dark. The nuclei were stained with 4′,6′-diamidino-2-phenylindole (DAPI, Invitrogen). Samples were analyzed by fluorescence microscopy (BX51, Olympus, Japan).

### Western blot analysis

Cellular protein was analyzed by Western blotting as reported [21]. Briefly, cellular protein was extracted with detergent, and the protein concentration of the lysates was determined for each sample with the BCA Protein Assay Kit (Beyotime, China). Equal amounts of protein were separated by 12% sodium dodecylsulfate-polyacrylamide gel electrophoresis (SDS-PAGE) and transferred to polyvinylidene difluoride (PVDF) membranes (Pierce Biotechnology, Inc. Rockford, IL, USA). After blocking with 5% skim milk, the PVDF membranes were incubated with p53, p21, cyclin E1, Fas-L, cleaved caspase 3,8,9 or β-actin primary antibody (all rabbit IgGs; 1∶1000; Cell Signaling Technology, Danvers, MA, USA) at 4°C overnight. The membranes were washed three times in PBST (10 mM NaH_2_PO_4_, 130 mM NaCl, 0.05% Tween 20) and then probed with a horseradish peroxidase-conjugated secondary antibody (goat anti rabbit IgG; 1∶5000; Molecular Probes, Invitrogen) for 1 h. Protein expression was visualized by the enhanced chemiluminescence (ECL) western blotting detection reagent (GE Health Care Bio-Sciences, Piscataway, NJ, USA).

### Surgical procedures

C57BL/6 mice (weight 22–26 g) (Institute of Model Animals, Nan Jing, China) were used in this study. The mice received a single right TBI induction with an electromagnetically controlled cortical impact device that allowed for the administration of precisely controlled cortical injuries, as previously described [22]. Briefly, mice were anesthetized with 4% isoflurane and a 1∶1 mixture of N_2_O/O_2_, and the head was mounted in a digital stereotaxic frame (David Kopf Instruments, Tujunga, California, USA). A midline incision was made, and a 5 mm craniotomy was performed over the right fronto-parietal cortex. A 3-mm impact tip was positioned over the center of the craniotomy site 3.0 mm anterior to lambda and 2.7 mm to the right of midline. TBI was triggered using a Matlab-based computer controller. An impact injury was delivered to compress the cortex to a depth of 1.0 mm at a velocity of 5 m/s for a 100 ms duration. Next, the craniotomy hole was sealed with bone wax while the scalp incision was fastened with a suture, and mice were allowed to recover on a heating pad before being returned to their home cages. Sham-injured mice (sham surgery group) received similar surgical procedures including a craniotomy but no TBI.

### NSC transplantation

TBI mice were then divided into a sham treatment group and multiple treatment groups. Treatment groups included the harvesting media-exposed NSC groups (Saline-exposed NSC, PBS-exposed NSC and ACSF-exposed NSC) and the PCM-exposed NSC group. Passage 3 NSCs from E14.5 EGFP-transgenic mice (EGFP-NSCs) were exposed to the harvesting media (Saline, PBS or ACSF) or the PCM alone for 6 h before transplantation. One week post-TBI, TBI mice were anesthetized and transplanted with a 5 µl suspension of treated NSCs (2×10^5^/µl) by in situ injection to 4 spots within the lesion sites adjacent to the cavity, using stereotactic guidance. Sham treatment mice that underwent identical procedures without any injection of cells and TBI mice that received mere the harvesting media or the PCM injection were employed as controls. Cyclosporine A (10 mg/kg) (Beyotime) was injected *i.p.* to suppress host immunologic rejection once per day for 7 days after grafting.

### Histopathological analyses

Twenty-one days after transplantation, the brains were dissected, post-fixed in 4% paraformaldehyde, equilibrated in 10%, 20% and 30% sucrose and sectioned on a freezing microtome (CM 1950, Leica) in 10-µm coronal slices and subjected to hematoxylin and eosin (HE) or immunohistochemical staining. For immunohistochemical staining, frozen sections of the brain were blocked using 5% BSA and 0.25% Triton X-100 for 1 h, incubated overnight at 4°C with anti-microtubule associated protein 2 (MAP2; rabbit IgG; 1∶200; Proteintech Group, Inc., Chicago, IL, USA), β-tubulin-III (rabbit IgG; 1∶200; Chemicon), synaptophysin (rabbit IgG; 1∶200; Sigma) or GFP primary antibodies (mouse IgG; 1∶200; Proteintech Group). All antibodies were diluted in Tris-buffered saline supplemented with 0.5% Tween 20 (TBST). After incubation with secondary antibody (goat anti-rabbit IgG-Alexa Fluor 594 or goat anti-mouse IgG-Alexa Fluor 488; 1∶5000; Molecular Probes, Invitrogen) for 20 min and counterstaining with DAPI (1∶500, Sigma-Aldrich) for 5 min at room temperature in the dark, sections were viewed using a fluorescence microscope (BX51, Olympus, Japan).

### Motor function testing

For the assessment of motor function deficit and recovery, the rotarod test and beam walk test were conducted. Animals were adapted to these tasks every second day starting 1 week before surgery. These motor function tests were performed on days 1, 3, 7, 10, 14, 17, 21, 24 and 28 post-TBI. For the rotarod test, mice were placed on a rotating rod (10 mm diameter) (Ugo Basile, Collegeville, PA). The rotational speed of the device was increased in increments of 3 rpm/5 sec, from 0 to 40 revolutions per minute (rpm). The maximal speed at which each mouse failed to stay on the rod was recorded. For the beam walk test, mice were placed on a narrow wooden beam 6 mm in width and 120 cm in length suspended 30 cm above the ground. When mouse walked across the beam, the number of foot faults for the left hindlimb was counted over 50 steps.

### Data analysis

For all studies, at least three independent experiments (n as detailed in the Results) were performed, and the data handling and statistical processing were performed using Microsoft Excel and GraphPad Prism software. Data are presented as the mean ± standard deviation (SD) or mean ± standard error of the mean (SEM). One-way or two-way analysis of variance (ANOVA) for repeated measures was used to determine statistically significant differences for all property tests. Bonferroni's post hoc test for multiple comparisons was used to determine data points with significant differences. *P* values<0.05 were statistically significant. All the images collected by optical or fluorescence microscopy were analyzed by Image Pro Plus 6.0 and processed using Adobe Photoshop.

## Results

### Effects on cell viability and proliferation

**Figure 1:**
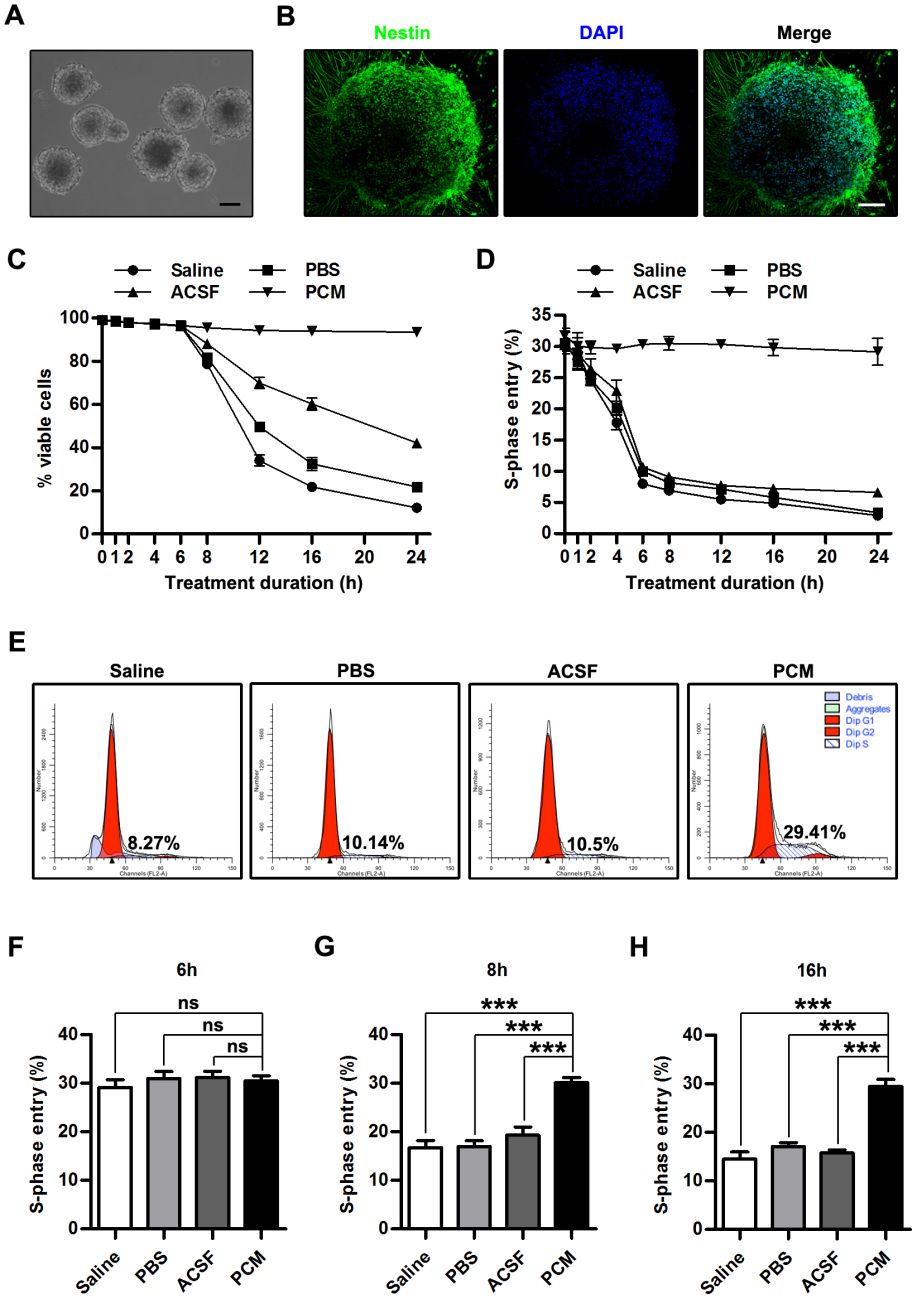
Effects on cell viability and proliferation. (A) Phase contrast image of neurospheres at passage 3 from the cerebral cortex of E14.5 C57BL/6 mice. (B) Immunofluorescence images of neurospheres stained for nestin and DAPI. (C) Quantitative analysis of the viability of NSCs exposed to the harvesting media or the PCM for 0, 1, 2, 4, 6, 8, 12, 16 or 24 h. (D) Quantitative analysis of S-phase entry (percentage of BrdU-positive cells) in dissociated NSCs exposed to the harvesting media or the PCM for 0, 1, 2, 4, 6, 8, 12, 16 or 24 h. (E) Images of cell cycle analysis by flow cytometry of NSCs exposed to the harvesting media or the PCM for 6 h. (F) Quantitative analysis of S-phase entry (percentage of BrdU-positive cells) in dissociated NSCs exposed to the harvesting media for 6 h and successively cultured in the PCM for another 24 h. (G) Quantitative analysis of S-phase entry (percentage of BrdU-positive cells) in dissociated NSCs exposed to the harvesting media for 8 h and successively cultured in the PCM for another 24 h. (H) Quantitative analysis of S-phase entry (percentage of BrdU-positive cells) in dissociated NSCs exposed to the harvesting media for 16 h and successively cultured in the PCM for another 24 h. For (C) and (D), data are presented as mean ±SEM; n = 5 per group per time point. For (F), (H) and (G), data are presented as mean ±SEM; ns, nonsignificant; ***, p<0.001; n = 5 per group. Scale bar: (A)  = 100 µm; (B)  = 50 µm. Abbreviation: BrdU, 5-bromo-2-deoxyuridine; DAPI, 4′,6′-diamidino-2-phenylindole; E14.5, embryonic day 14.5; NSC, neural stem cell; PCM, proliferation culture medium; SEM, standard error of the mean.

To study the effects of the various harvesting media on NSCs, we first isolated NSCs from the cerebral cortex of E14.5 mice. As shown in Fig. 1A, dissociated single cells formed neurospheres after 5–7 days in the PCM consisting of DMEM/F-12 supplemented with EGF and bFGF. Neurospheres showed nestin-positive immunoreactivity (Fig. 1B). Then we examined the effects of harvesting media exposure on the viability of NSCs in culture by trypan blue exclusion assay. As shown in Fig. 1C, no obvious difference was detected in the cell viability between the harvesting media (Saline, PBS or ACSF) and the PCM groups when exposure time was shorter than 6 h (Saline, PBS and ACSF, p>0.05 versus PCM on 0, 1, 2, 4 and 6 h; n = 5 per group per time point). However, as treatment was prolonged longer than 8 h, harvesting media exposure resulted in a significant decrease in viable cell in contrast to exposure to PCM (Saline, PBS and ACSF, p<0.001 versus PCM on 8, 12, 16 and 24 h; n = 5 per group per time point) and this decrease occurred in a time-dependent manner (p<0.001; n = 5 per group per time point). Additionally, when NSCs were exposed to the harvesting media for more than 8 h, the proportion of viable cells was lowest in the Saline treatment group, followed in order by the PBS group, and the ACSF group (ACSF, p<0.001 versus Saline or PBS on 8, 12, 16 and 24 h; PBS, p<0.001 versus Saline on 12, 16 and 24 h n = 5 per group per time point). In accordance with the protocol of the US Food and Drug Administration for human somatic cell therapy, the viability of the cells should be quantitated and a lower limit of acceptability established [23]. In the current study, the permissible limit was established at 70%. Harvesting media exposure elicited an almost 30% reduction in the viability of cells in 8 to 12 h (at approximately 9 h for Saline, 9.5 h for PBS and 12 h for ACSF).

Factors reducing cell viability and inducing cell death may also attenuate cellular proliferation and cell cycle progression [20]. To address whether cell cycle progression is affected by exposure to the harvesting media, we examined S-phase entry in either the harvesting media or the PCM exposed- NSCs by BrdU incorporation assay (Fig. 1D). Inconsistent with the results from cell viability assay, harvesting media-exposed cells displayed significantly reduced proliferation only after 2-hour treatment compared with PCM-exposed cells and ratio of S-phase entry in harvesting media exposure group decreased rapidly in the first 6 hours and thenceforth relatively slowly (Saline and PBS, p<0.01 versus PCM on 2 h; Saline, PBS and ACSF, p<0.001 versus PCM on 4, 6, 8, 12, 16 and 24 h; n = 5 per group per time point). Similar to the decrease in cell viability, the reduction of proliferation also showed a time-dependent manner (p<0.001; n = 5 per group per time point; Fig. 1D). After exposure to harvesting media for 6 h, the NSCs on S-phase decreased as much as two-thirds (Saline  = 7.98±0.23%; PBS = 10.00±0.11%; ACSF = 10.66±0.30%; PCM = 30.33±0.49%; mean ±SEM; n = 5 per group), which was also revealed by flow cytometry analysis (Fig. 1E). However, during the continued decline of NSC proliferation activity, almost no difference was observed among ACSF, Saline and PBS-exposed cells (ACSF, p>0.05 versus Saline or PBS on 0, 1, 2, 6, 8, 12, 16, 20 and 24 h; PBS, p>0.05 versus Saline on 0, 1, 2, 4, 6, 8, 12, 16, 20 and 24 h n = 5 per group per time point). Interestingly, after the NSCs exposed to the harvesting media for 6 h were transferred to the PCM and cultured for another 24 h, the proliferation were restored to the levels equivalent to the cells exposed to the PCM (Saline  = 29.12±1.69%; PBS = 30.98±1.43%; ACSF = 31.14±1.30%; PCM = 30.46±1.07; Saline, PBS and ACSF, p>0.05 versus PCM; mean ±SEM; n = 5 per group; Fig. 1F). However, when NSCs that had underwent exposure to the harvesting media for more than 8 h were transferred to the PCM and cultured for 24 h, the impaired proliferation was not restored to the original (pre-treatment) levels with no evident difference among harvesting media exposure groups (For 8 h: Saline  = 16.74±1.48%; PBS = 16.99±1.16%; ACSF = 19.30±1.30%; PCM = 30.12±1.05%; Saline, PBS and ACSF, p<0.001 versus PCM; ACSF, p>0.05 versus Saline or PBS; PBS, p>0.05 versus Saline; mean ±SEM; n = 5 per group. For 16 h: Saline  = 14.52±1.42%; PBS = 17.07±0.81%; ACSF = 15.75±0.69%; PCM = 29.48±1.39%; Saline, PBS and ACSF, p<0.001 versus PCM; ACSF, p>0.05 versus Saline or PBS; PBS, p>0.05 versus Saline; mean ±SEM; n = 5 per group. Fig. 1G,H).

**Figure 2:**
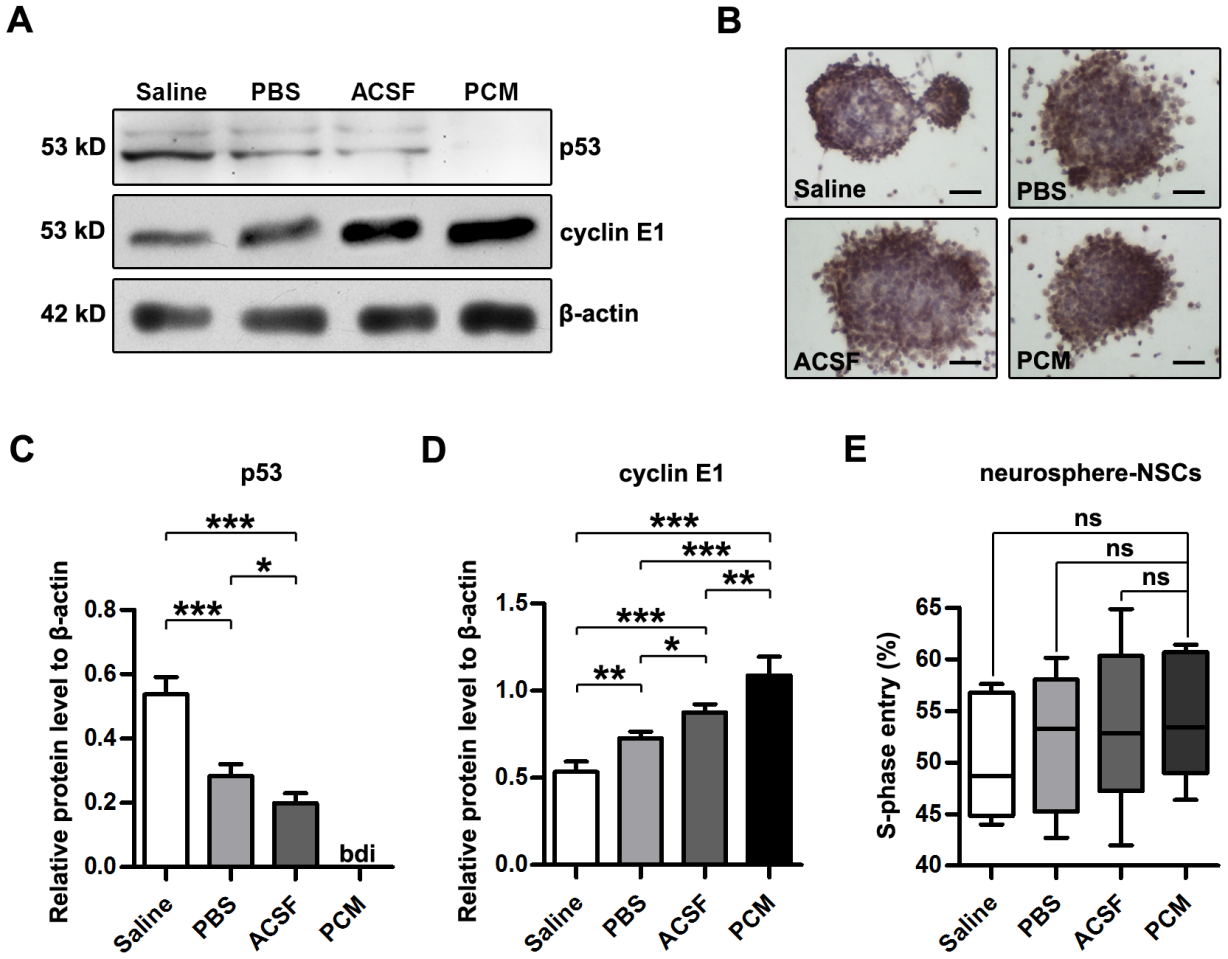
Mechanism of harvesting media exposure-induced proliferation inhibition. (A) Western blots of p53 and cyclin E1 from NSCs exposed to the harvesting media or the PCM for 6 h. β-actin was used as a loading control. (B) Images of BrdU incorporation assays of NSC-neurospheres exposed to the harvesting media or the PCM for 1 week. (C–D) Quantitative analysis of the relative levels of p53 (C) and cyclin E1 (D) to β-actin in NSCs exposed to the harvesting media or the PCM for 6 h. Data are presented as mean ±SD. n = 5 per group. bdi, below detectable limit; *, p<0.05; **, p<0.01; ***, p<0.001 versus PCM. (E) Quantitative analysis of S-phase entry (percentage of BrdU-positive cells) of NSCs in neurospheres exposed to the harvesting media or the PCM for 1 week. The box plots have Min to Max whiskers, a line for the median, and edges for the minimum and maximum. ns, nonsignificant; n = 5 per group. Scale bar: (B)  = 100 µm. Abbreviation: bdi, below detectable limit;BrdU, 5-bromo-2-deoxyuridine; NSC, neural stem cell; PCM, proliferation culture media; SD, standard deviation; SEM, standard error of the mean.

To elucidate the mechanism by which harvesting media exposure inhibited proliferation of NSCs, we examined the levels of p53, p21 and cyclin E1, which are related to the G_1_/S transition and cell cycle progression, using Western blot analysis. As shown in Fig. 2A,C,D, exposure to the harvesting media increased p53 protein (PCM, below detectable limit; Saline, PBS and ACSF, p<0.001 versus PCM; n = 5 per group) and reduced cyclin E1 protein (Saline, PBS and ACSF, p<0.001 versus PCM; n = 5 per group). However, p21, which acts downstream of p53, was below the detectable limit (data not shown), most likely due to its absence in embryonic stem cells [24], [25]. These results suggest that harvesting media exposure-induced proliferation inhibition and cell-cycle arrest may occur through upregulation of p53 and downregulation of cyclin E1. Of note, among the harvesting media-exposed NSCs, ACSF-exposed were detected with lowest p53 level and highest cyclin E1 expression while Saline-exposed NSCs with highest p53 level and lowest cyclin E1 expression (For p53, ACSF, p<0.05 versus PBS, p<0.001 versus Saline; PBS, p<0.001 versus Saline. For cyclin E1, ACSF, p<0.05 versus PBS, p<0.001 versus Saline; PBS, p<0.01 versus Saline. n = 5 per group). These data suggest that NSCs showed different alteration of p53 and cyclin E1 levels in response to a solution containing glucose or pH buffer system or not. The conflict that NSCs exposed to these different harvesting media displayed consistent proliferation inhibition but distinct molecular responses further illustrates that a constrained level of p53 and cyclin E1 is necessary to support normal cell growth [20], [26], [27].

Although the proliferation of dissociated NSCs was obviously attenuated by harvesting media exposure, NSCs in neurospheres remained highly proliferative even after 1-week exposure to the harvesting media (Saline  = 50.40±2.72%; PBS = 51.96±3.06%; ACSF = 53.60±3.66%; PCM = 54.56±2.76%; Saline, PBS and ACSF, p>0.05 versus PCM; n = 5 per group; Fig. 2B,E), which suggests a difference in the proliferation potential between dissociated NSCs and NSC-neurospheres.

### Effects on the apoptosis and necrosis of NSCs

**Figure 3:**
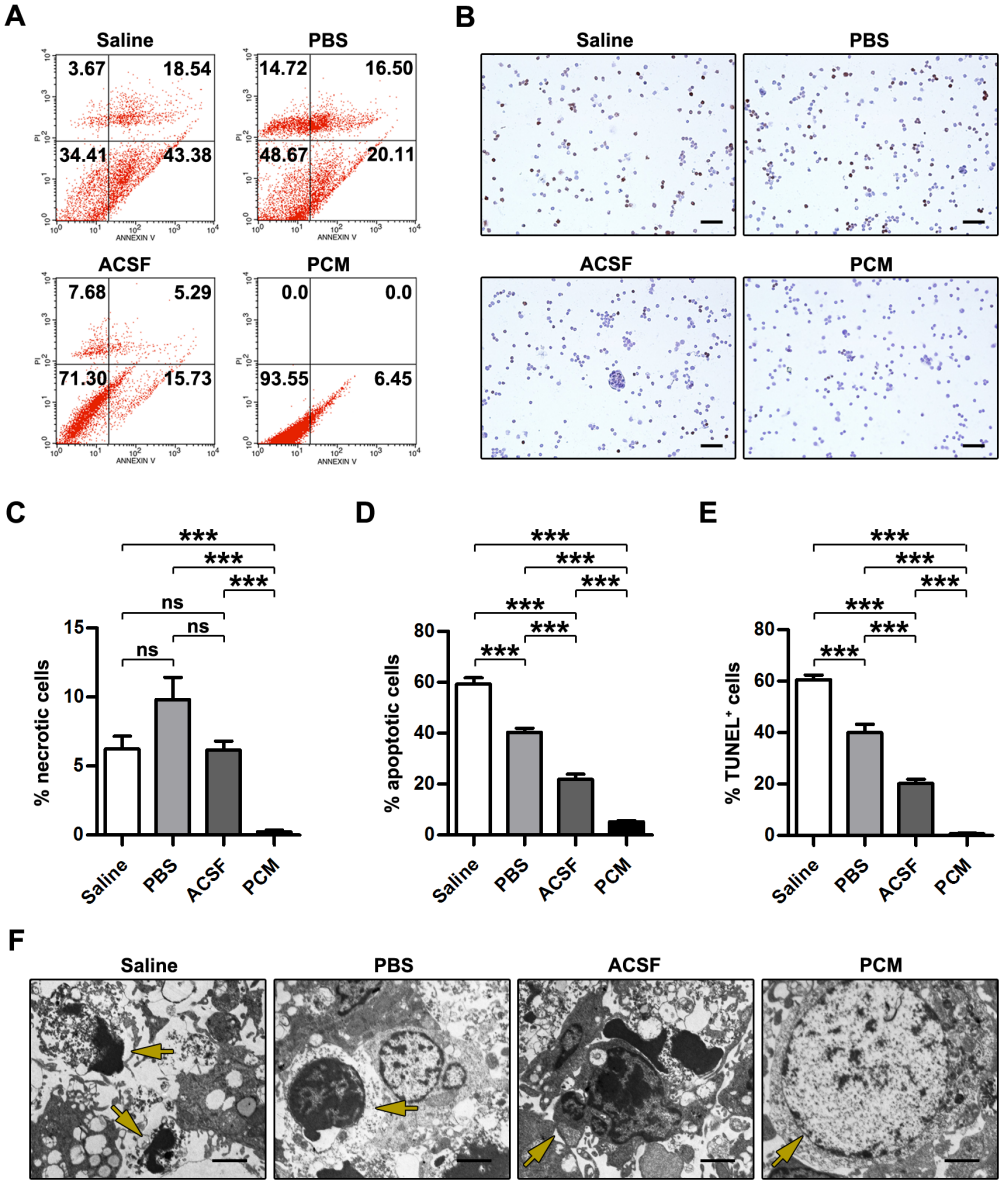
Effects on apoptosis and necrosis of NSCs. (A) Images of flow cytometry analysis of Annexin-V and PI staining of NSCs exposed to the harvesting media or the PCM for 12 h. (B) Images of TUNEL staining of NSCs exposed to the harvesting media or the PCM for 16 h. (C–D) Quantitative analysis of the percentage of apoptotic (C) and necrotic cells (D) in NSCs exposed to the harvesting media or the PCM for 12 h. Data are presented as mean ±SEM. ***, p<0.001 versus PCM. n = 5 per group. (E) Quantitative analysis of TUNEL-positive NSCs exposed to the harvesting media or the PCM for 16 h. Data are presented as mean ±SEM. ns, nonsignificant; ***, p<0.001 versus PCM. n = 10 per group. (F) TEM images of NSCs exposed to the harvesting media or the PCM for 12 h. For Saline, arrow denotes a loss of cytoplasm and evident karyopyknosis and nuclear fragmentation; for PBS, arrow denotes a loss of cytoplasm and evident karyopyknosis; for ACSF, arrow denotes apoptotic bodies and a loss of cytoplasm; for PCM, arrow denotes an intact nuclear envelope and uniformly dispersed chromatin. Scale bar: (C)  = 100 µm; (F)  = 1 µm. Abbreviation: NSC, neural stem cell; PCM, proliferation culture medium; PI, propidium iodide; SEM, standard error of the mean; TEM, transmission electron microscopy; TUNEL, terminal deoxynucleotidyl transferase (TdT) dilutions of cell-delivered dUTP nick 3′-end DNA labeling.

As revealed by the data from the cell viability assay (Fig. 1C), evident cell death occurred mainly after 8 to 16-hour exposure to the harvesting media. To further investigate the harvesting media exposure- induced cell death, the NSCs exposed to the harvesting media for 12 h were subjected to flow cytometry analysis. In contrast to PCM-exposed cells, the harvesting media-exposed cells displayed significant necrosis (Saline  = 6.24±0.93%; PBS = 9.82±1.60%; ACSF = 6.16±0.66%; PCM = 0.25±0.12%; Saline, PBS and ACSF, p<0.001 versus PCM; mean ±SEM; n = 5 per group; Fig. 3A,C) as well as apoptosis including early and late apoptosis (Saline  = 59.25±2.51%; PBS = 40.35±1.67%; ACSF = 21.89±1.97%; PCM = 5.20±0.47%; Saline, PBS and ACSF, p<0.001 versus PCM; mean±SEM; n = 5 per group; Fig. 3A,D). No significant difference in necrosis was detected among Saline, PBS and ACSF-exposed cells (ACSF, p>0.05 versus Saline or PBS; PBS, p>0.05 versus Saline; n = 5 per group). However, highest percentage of apoptotic cells existed in Saline exposure group whereas fewer PBS-exposed cells and fewest ACSF-exposed cells underwent apoptosis (Saline, p<0.001 versus PBS or ACSF; PBS, p<0.001 versus ACSF; n = 5 per group).

To investigate DNA fragmentation of the apoptotic NSCs induced by harvesting media exposure, we examined NSCs exposed to the harvesting media for 16 h by the TUNEL assay. Coinciding with the FCM data, the results of TUNEL assay showed more TUNEL-positive apoptotic cells in the harvesting media exposure groups (Saline  = 60.60±1.93%; PBS = 40.00±3.21%; ACSF = 20.17±1.73%; PCM = 0.64±0.29%; Saline, PBS and ACSF, p<0.001 versus PCM; mean ±SEM; n = 10 per group; Fig. 3B,E). Further, ACSF which contains buffer system and glucose displayed best apoptosis-protective property whereas Saline-exposed NSCs were most vulnerable to apoptosis (Saline, p<0.001 versus PBS or ACSF; PBS, p<0.001 versus ACSF; n = 10 per group). These findings in FCM and TUNEL analysis suggested that exposure to the harvesting media triggered both necrosis and apoptosis of NSCs. Furthermore, NSC necrosis posterior to harvesting media exposure showed random fluctuation whereas harvesting media exposure-associated apoptosis was concerned with components of solutions.

To visualize the morphological changes of apoptotic NSCs, we performed TEM. Under TEM, NSCs showed normal morphological features in the PCM treatment group. The cell membrane and nuclear envelope were intact, and the nuclear chromatin was uniformly dispersed. By contrast, the cells exposed to the harvesting media for more than 12 h showed features of apoptotic cells, including a reduction and loss of cellular processes, formation of apoptotic bodies, loss of cytoplasm, karyopyknosis and nuclear fragmentation (Fig. 3F).

**Figure 4:**
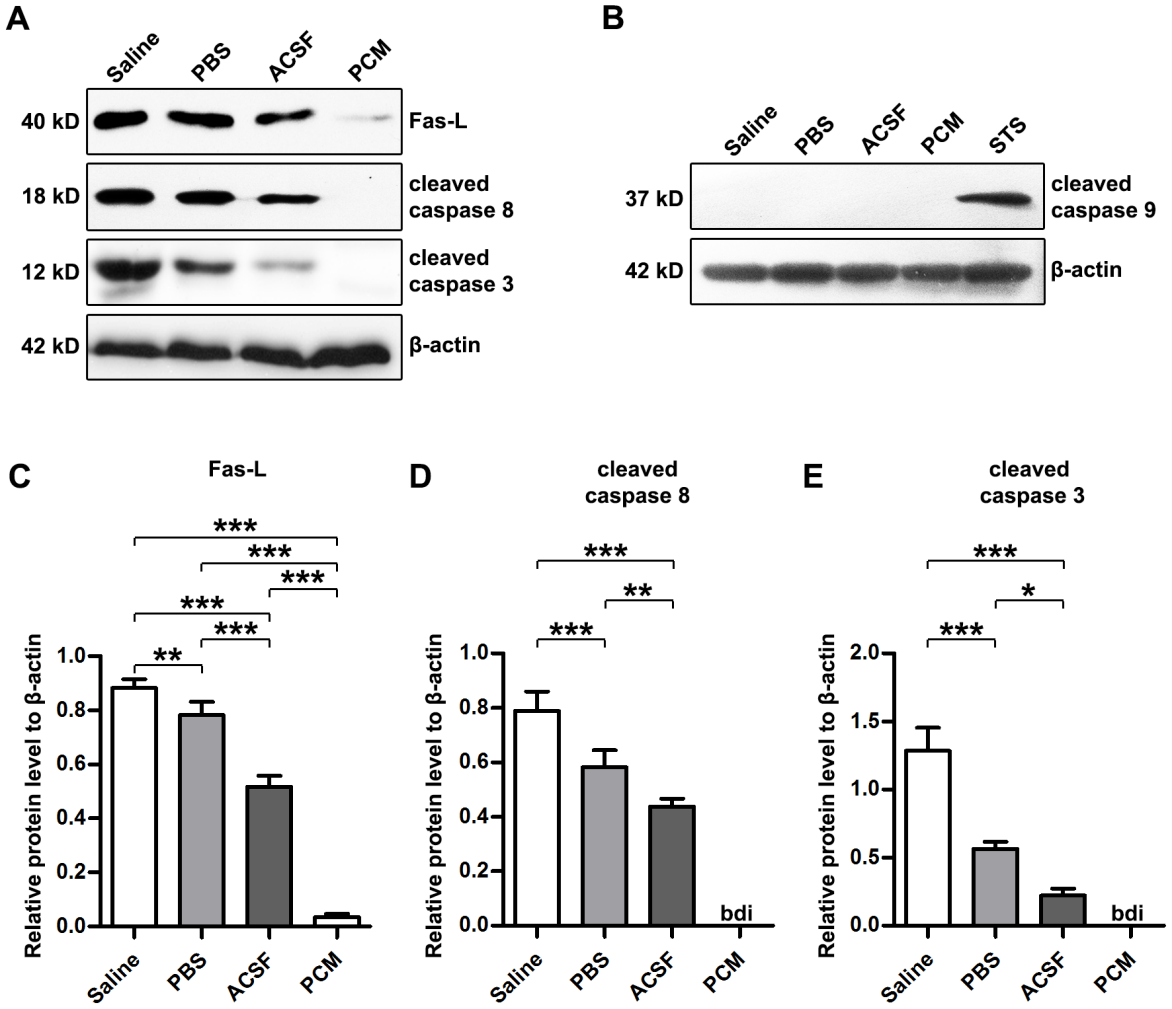
Western blot analysis of apoptosis-related molecules in harvesting media-exposed NSCs. (A) Western blots of Fas-L, cleaved caspase 8 and 3 from NSCs exposed to the harvesting media or the PCM for 12 h. β-actin was used as a loading control. (B) Western blots of cleaved caspase 9 from NSCs exposed to the harvesting media, the PCM or 5 µM staurosporine (positive control) for 12 h. β-actin was used as a loading control. (C–D) Quantitative analysis of the levels of Fas-L (C), cleaved caspase 3 (D) and cleaved caspase 8 (E) relative to β-actin in NSCs exposed to the harvesting media or the PCM for 12 h. Data are presented as mean ±SD. n = 5 per group. bdi, below detectable limit; *, p<0.05; **, p<0.01; ***, p<0.001 versus PCM. Abbreviation: ACSF, artificial cerebrospinal fluid; bdi, below detectable limit; NSC, neural stem cell; PCM, proliferation culture medium; SD, standard deviation; STS, staurosporine.

**Figure 5:**
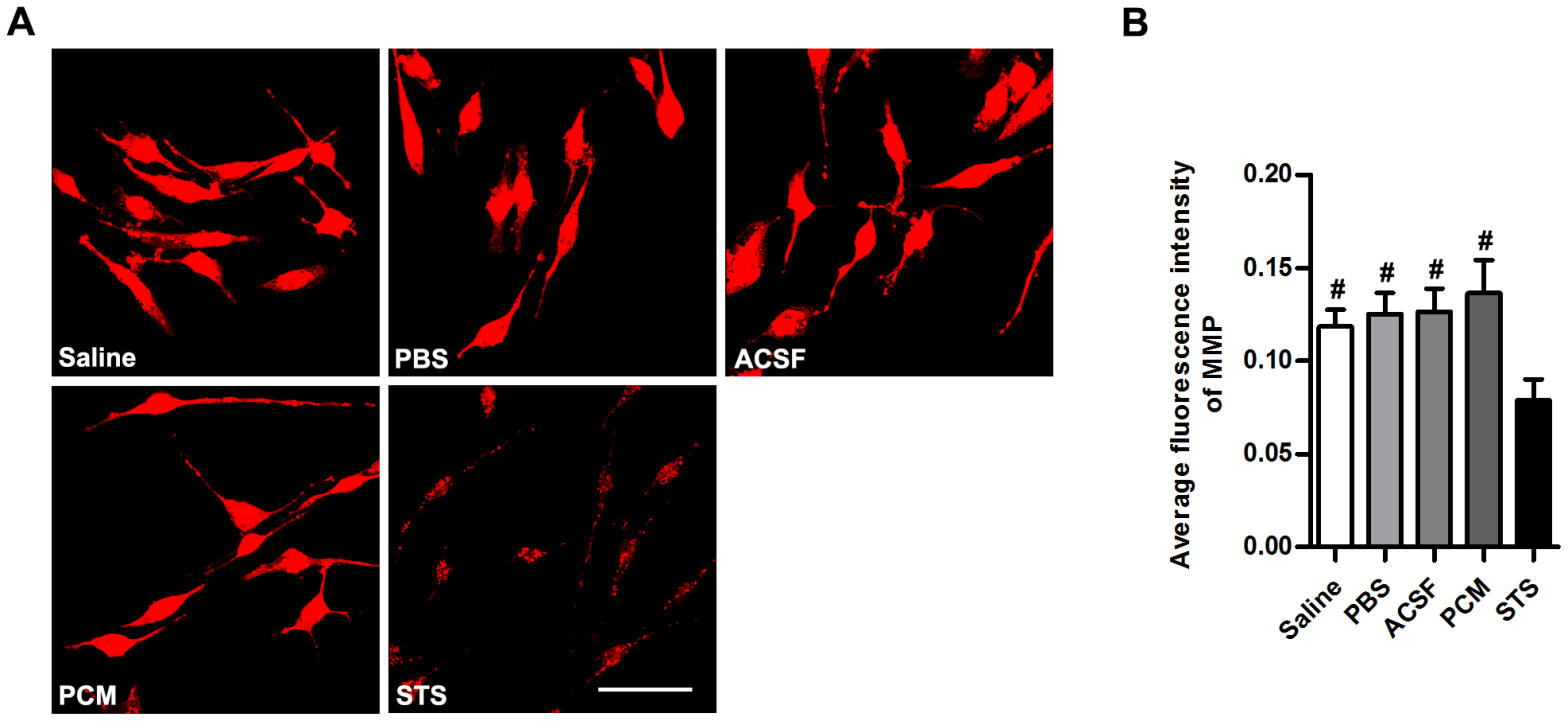
MMP analysis of harvesting media-exposed NSCs. (A) Confocal images of dissociated NSCs exposed to the harvesting media, the PCM or 5 µM staurosporine (positive control) for 12 h and stained for MMP. (B) Quantitative analysis of average fluorescence intensity of MMP of NSCs exposed to the harvesting media, the PCM or 5 µM staurosporine (positive control) for 12 h. Data are presented as mean ±SD; #, p<0.001 versus STS; ns, nonsignificant versus STS; n = 5 per group. No significant difference was detected between the harvesting media-exposed NSCs and PCM-exposed NSCs. Scale bar: (A)  = 100 µm. Abbreviation: ACSF, artificial cerebrospinal fluid; MMP, mitochondrial membrane potential; NSC, neural stem cell; PCM, proliferation culture medium; SD, standard deviation; STS, staurosporine.

To further unravel the molecular mechanisms mediating apoptosis in these cells, we measured the expression of several apoptosis-related molecules by Western blot analysis. As shown in Fig. 4A, C–E, increased Fas-L expression (Saline, PBS and ACSF, p<0.001 versus PCM; n = 5 per group) coupled with elevated levels of cleaved caspase 8 (PCM, below detectable limit; Saline, PBS and ACSF, p<0.001 versus PCM; n = 5 per group) and cleaved caspase 3 (PCM, below detectable limit; Saline, PBS and ACSF, p<0.001 versus PCM; n = 5 per group) were observed in NSCs in the experimental groups. Consistent with proportions of apoptotic cells, among the harvesting media exposure groups, ACSF-exposed cells showed the lowest expression of these apoptosis-related molecules which was highest in NSCs exposed to Saline (For Fas-L: ACSF, p<0.001 versus Saline or PBS; PBS, p<0.01 versus Saline; n = 5 per group. For cleaved caspase 8: ACSF, p<0.001 versus Saline, p<0.01 versus PBS; PBS, p<0.001 versus Saline; n = 5 per group. For cleaved caspase 3: ACSF, p<0.001 versus Saline, p<0.05 versus PBS; PBS p<0.001 versus Saline; n = 5 per group). In contrast to Fas-L and activated caspase 8, cleaved caspase 9 expression remained undetectable (Fig. 4B), suggesting the involvement of Fas-L/Fas-caspase 8-caspase 3 axis (extrinsic pathway) activation in the apoptosis induced by harvesting media exposure. To further determine whether the intrinsic pathway implicated, the mitochondrial membrane potential (MMP) in harvesting media-exposed NSCs was assayed (Fig. 5). The results showed equivalent average fluorescence intensity of MMP (Saline, PBS and ACSF, p>0.05 versus PCM; n = 5 per group) in either the harvesting media or the PCM-exposed NSCs. The unaltered MMP and undetected activated caspase 9 indicated that the mitochondrial inner membrane was relatively intact and excluded the intrinsic pathway activation in NSCs exposed to the harvesting media.

Together, our results indicate that obvious NSC apoptosis and necrosis occurred following exposure to the harvesting media for longer than 8 h. Harvesting media exposure-associated NSC apoptosis was mediated by the extrinsic rather than intrinsic signaling pathways.

### Effects on cell differentiation

**Figure 6:**
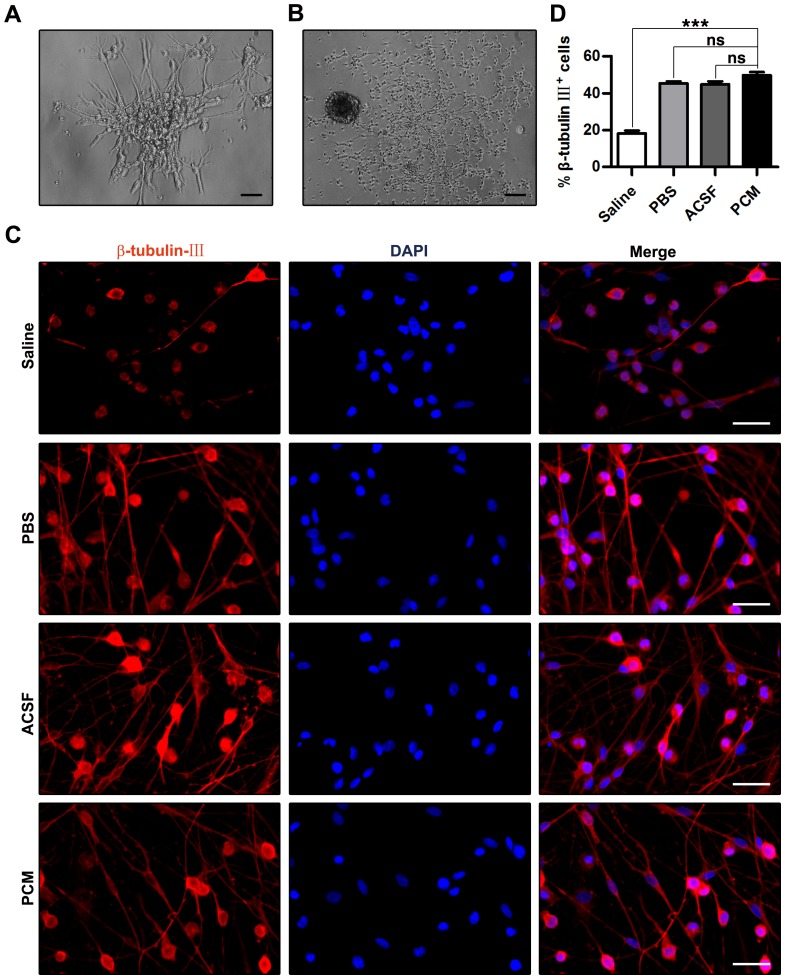
Effects on cell differentiation. (A) Phase contrast image of NSCs cultured in the differentiation medium for 7 days. Cells with neurite-like projections migrated from the edge of neurospheres. (B) Phase contrast image of differentiated cells with bipolar, tri-polar or multipolar processes migrating from neurospheres. (C) Fluorescence images of NSCs exposed to the harvesting media for 6 h, differentiated for 14 days and stained with anti-β-tubulin-III polyclonal antibody and counterstained with DAPI. (D) Quantitative analysis of β-tubulin-III-positive cells in NSCs exposed to the harvesting media for 6 h and differentiated for 14 days. Data are presented as mean ±SEM. n = 10 per group. ns, nonsignificant versus PCM; ***, p<0.001 versus PCM. Scale bar: (A)  = 100 µm; (B)  = 200 µm; (C)  = 25 µm. Abbreviation: DAPI, 4′,6′-diamidino-2-phenylindole; NSC, neural stem cell; SEM, standard error of the mean.

To determine whether isolated NSCs have the ability to differentiate, we first transferred neurospheres to poly-D-lysine and laminin-coated coverslips and cultured them in the differentiation medium for 14 days. As shown in Fig. 6A, after 7 days in culture, some cells with neural projections migrated from the neurospheres. After 10 days in culture (Fig. 6B), differentiated cells with bipolar, tri-polar or multipolar processes were generated from the neurospheres. To investigate whether harvesting media exposure regulated NSC fate determination, cultured NSCs dissociated enzymatically and exposed to the various harvesting media for 6 h which exhibited obviously attenuated proliferation rather than cell viability, were then reseeded onto poly-D-lysine and laminin-coated coverslips in 24-well plates containing the differentiation medium for 7–14 days. As shown in Fig. 6C,D, there was no significant difference in the percentage of β-tubulin III+ cells between the PBS or ACSF group and the PCM group (PBS = 45.30±1.21%; ACSF = 44.85±1.62%; PCM = 49.80%±1.63%; PBS and ACSF, p>0.05 versus PCM; mean ±SEM; n = 10 per group). However, Saline exposure resulted in a marked decrease in the percentage of β-tubulin III+ cells (Saline  = 18.27%±1.50%; Saline, p<0.001 versus PCM; mean ±SEM; n = 10 per group) compared with PCM exposure. When neurospheres which underwent 1-week harvesting media exposure and showed resistance to proliferation inhibition after exposure to the harvesting media were subjected to the differentiation assay, the treated neurospheres, even the Saline-exposed neurospheres displayed high neuronal development potency (up to 75∼78%) with no evident differentiation impairment observed (Saline  = 73.06±2.89%; PBS = 75.37±3.27%; ACSF = 76.24±2.04%; PCM = 78.08±2.68%; Saline, PBS and ACSF, p>0.05 versus PCM; mean ±SEM; n = 10 per group; Fig. S1). These results indicate that exposure to PBS and ACSF has no significant influence on NSC differentiation into terminal neural cells, while Saline exposure impairs the *in vitro* differentiation of NSCs to neurons. Additionally, neurospheres are protected from Saline exposure-induced *in vitro* neurogenesis deterioration.

### Effects on the survival of grafted NSCs

**Figure 7:**
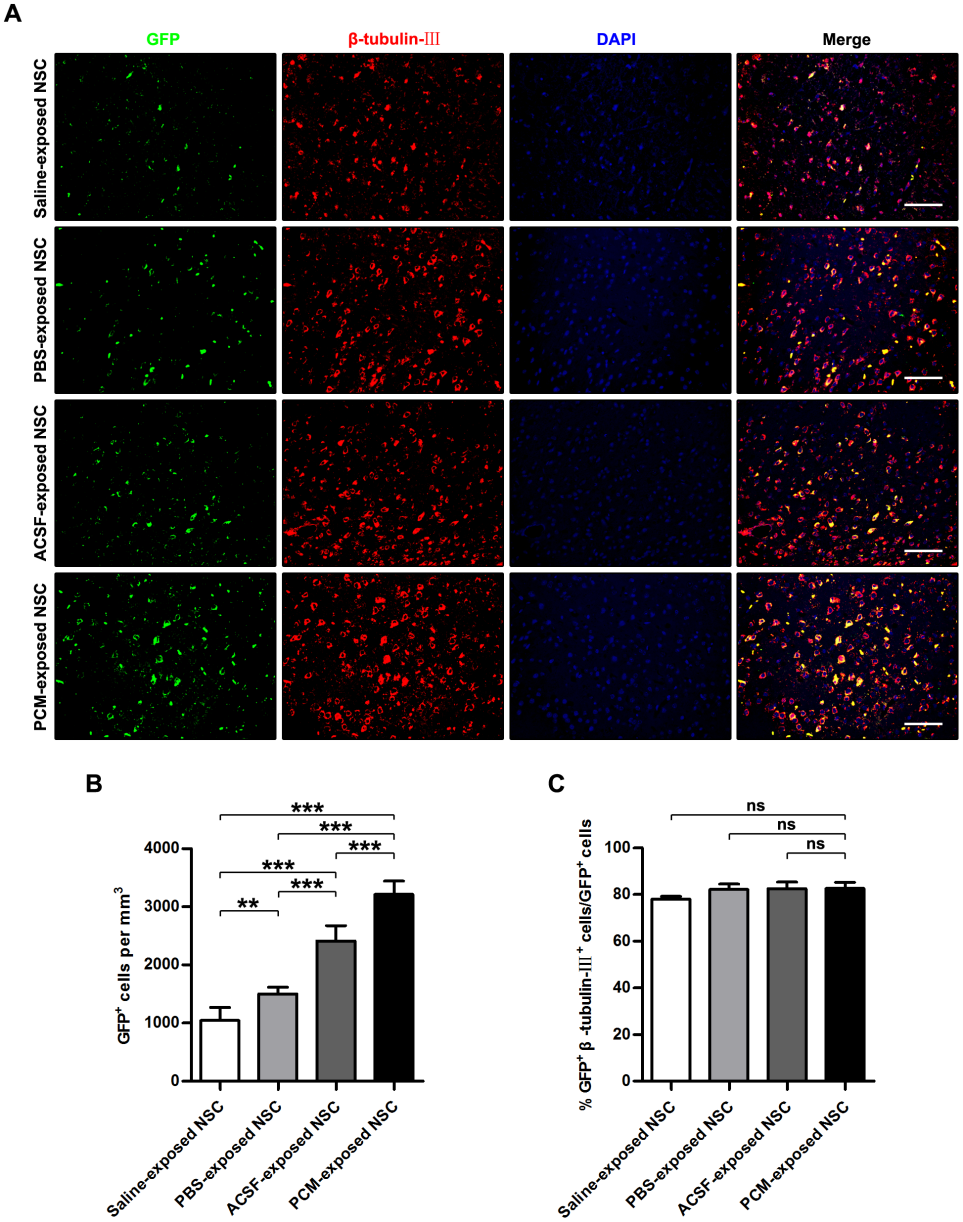
Effects on the survival and in vivo differentiation of grafted NSCs. (A) Immunofluorescence images of TBI murine cerebral cortex receiving the harvesting media or PCM-exposed NSC grafting for 21 days and stained for GFP and β-tubulin-III and counterstained by DAPI. (B–C) Quantitative analysis of survival (B) and in vivo differentiation (C) of grafted NSCs in host tissue. Data are presented as mean ±SEM; **, p<0.01; ***, p<0.001; ns, nonsignificant; n = 6 per group. Scale bar: (A)  = 50 µm. Abbreviation: DAPI, 4′,6′-diamidino-2-phenylindole; GFP, green fluorescence protein; NSC, neural stem cell; PCM, proliferation culture medium; SEM, standard error of deviation; TBI, traumatic brain injury; TEM, transmission electron microscopy.

**Figure 8:**
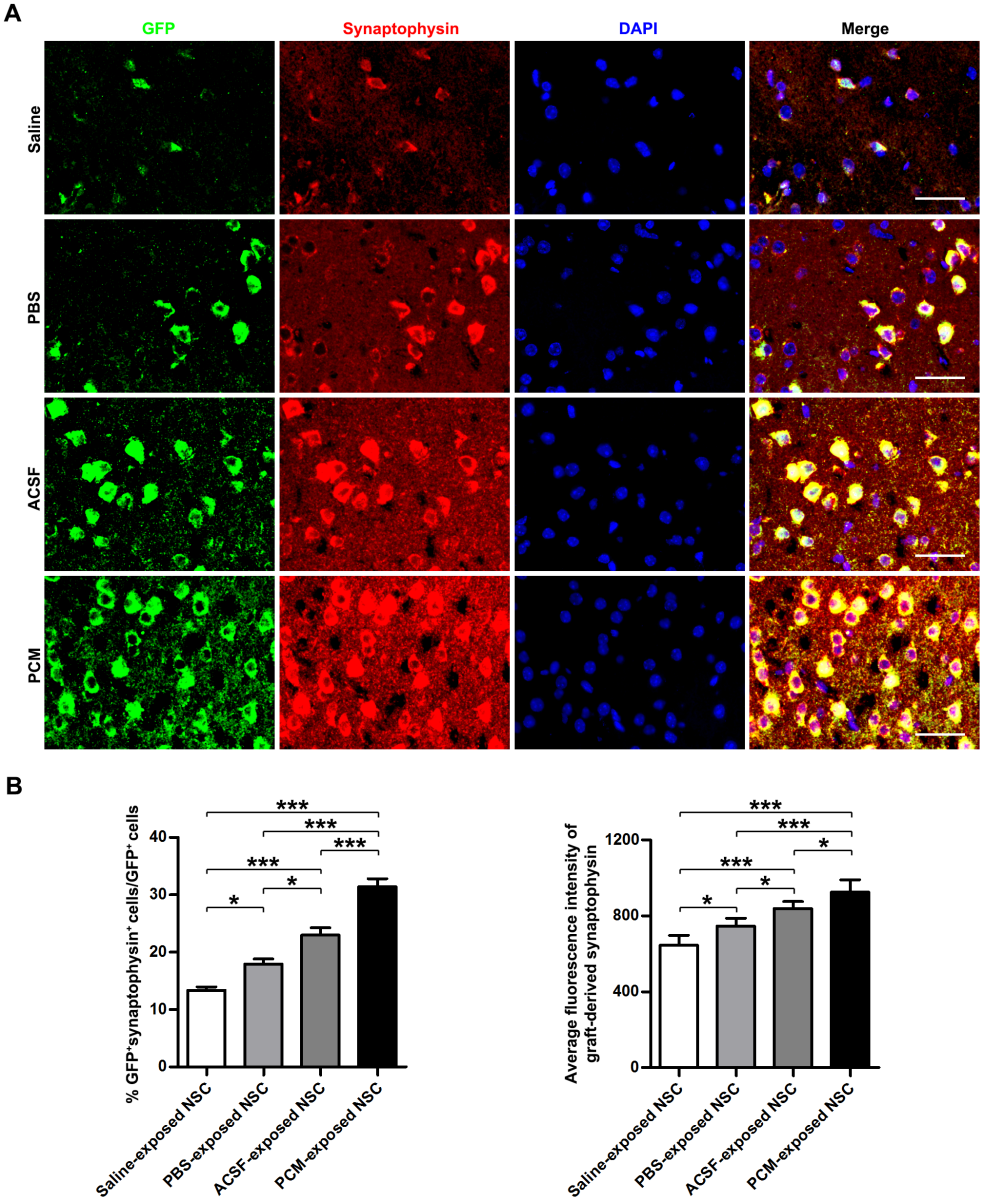
Effects on the synaptogenesis by graft-derived cells. (A) Immunofluorescence images of TBI murine cerebral cortex receiving the harvesting media or PCM-exposed NSC grafting for 21 days and stained for GFP and synaptophysin. (B) Quantitative analysis of percentage of synaptophysin-positive cells in GFP-positive graft-derived cells.. Data are presented as mean ±SEM; *, p<0.05; ***, p<0.001. (C) Quantitative analysis of fluorescence intensity of graft-derived synaptophysin. Data are presented as mean ±SD; ***, p<0.001. Scale bar: (A)  = 25 µm. Abbreviation: GFP, green fluorescence protein; NSC, neural stem cell; PCM, proliferation culture medium; SD, standard deviation; SEM, standard error of deviation; TBI, traumatic brain injury.

Since pre-grafting processes such as delivery and storage last long and 6-hour exposure to the harvesting media gave rise to notable proliferation inhibition rather than cell death, NSCs exposed to the harvesting media for 6 h were grafted *in situ* to the lesion sites of C57BL/6 mice with TBI. To investigate the effects of the harvesting media exposure on the survival and integration of transplanted NSCs derived from EGFP-transgenic mice, we performed morphological analyses. As observed by HE-stained sections, TBI led to a loss of cortical tissue and a distortion in the morphology of the remaining cortex (Fig. S2A), which was shown by TEM as hemorrhage, neuronal necrosis, neuronal apoptosis and phagocytosis by microglia in the injured cortical area (Fig. S2C). Immunohistochemical staining of the cortex in lesion sites also displayed neuron loss, arrangement disorder of remaining neurons and cortical structural damage caused by TBI (Fig. S2B). Besides, transplanted cells were evidenced by immunohistochemistry to survive within lesion sites for 21 days post-grafting (Fig. 7A). Occasionally, a few graft-derived cells were also found outside the injury area. However, significantly fewer green fluorescence protein (GFP)-labeled grafted cells that had been exposed to Saline, PBS or ACSF survived in the host tissue than cells exposed to the PCM (Saline-exposed NSC = 1043±225.7/mm^3^; PBS-exposed NSC = 1501±116.0/mm^3^; ACSF-exposed NSC = 2414±260.1/mm^3^; PCM-exposed NSC = 3212±229.1/mm^3^; Saline-exposed NSC, PBS-exposed NSC and ACSF-exposed NSC, p<0.001 versus PCM-exposed NSC; mean ±SD; n = 6 per group; Fig. 7A,B). The declined survival of grafted cells in harvesting media-exposed NSC treatment groups paralleled the stalled morphological restoration. Among the harvesting media-exposed NSC treatment groups, most GFP^+^ cells were detected in the cortical tissues of TBI mice that received ACSF-exposed NSC grafting whereas fewer PBS-exposed NSCs and the fewest Saline-exposed NSCs survived in the host tissue (ACSF-exposed NSC, p<0.001 versus Saline-exposed NSC or PBS-exposed NSC; PBS, p<0.01 versus Saline-exposed NSC; n = 6 per group), which accorded with the *in vitro* survival and proliferation assay and further confirmed the partial protective effect of glucose and buffer system in the harvesting media. Interestingly, the neuronal differentiation efficiency of grafted cells in the harvesting media-exposed NSC treatment groups (even the Saline-exposed NSC treatment group) were comparatively unaffected (Saline-exposed NSC = 78.02±1.16%; PBS-exposed NSC = 82.12±2.40%; ACSF-exposed NSC = 82.46±2.91%; PCM-exposed NSC = 82.63±2.62%; Saline-exposed NSC, PBS-exposed NSC and ACSF-exposed NSC, p>0.05 versus PCM-exposed NSC; n = 6 per group; Fig. 7A,C). This result indicated that the reduced repair potential was independent of the differentiation potency alteration. The contradiction between *in vivo* and *in vitro* differentiation of Saline-exposed NSCs may result from the complex milieu of the CNS different from the defined differentiation medium. Synapse formation is considered as not only the indicator of integration of grafted neural cells into host tissue but also the structural basis of graft-mediated recovery [28], [29]. As shown by synaptophysin (the specific marker of synaptic vesicle) staining, lower proportions of cells derived from harvesting media-exposed NSCs were detected with synaptophysin expression in host tissue when compared with cells differentiated from PCM-exposed NSCs (Saline-exposed NSC: 13.38±0.59%; PBS-exposed NSC: 17.93±0.91%; ACSF-exposed NSC: 22.97±1.30%; PCM-exposed NSC: 31.44±1.39%; Saline-exposed NSC, PBS-exposed NSC and ACSF-exposed NSC, p<0.001 versus PCM-exposed NSC; n = 6 per group). Among the harvesting media-exposed NSC groups, ACSF-exposed NSC group had the highest percentages of GFP-positive cells expressing synaptophysin, whereas PBS-exposed NSC group had lower and Saline-exposed NSC group had the lowest fraction of GFP^+^/synaptophysin^+^ cells (ACSF-exposed NSC, p<0.001 versus Saline-exposed NSC, p<0.05 versus PBS-exposed NSC; PBS, p<0.05 versus Saline-exposed NSC; n = 6 per group). Additionally, the average synaptogenesis of graft-derived neurons (calculated as the average fluorescence intensity of synaptophysin in GFP-labelled cells) was evidently reduced by harvesting media exposure prior to transplantation (Saline-exposed NSC and PBS-exposed NSC, p<0.001 versus PCM-exposed NSC; ACSF-exposed NSC, p<0.05 versus PCM-exposed NSC; n = 6 per group; Fig. 8A,C). Thereinto, the synapse formation of individual neuron derived from ASCF-exposed NSC was affected to the least extent. In contrast, progenies of PBS-exposed NSC showed moderate reduction of synapse formation and the most serious impairment of synaptogenesis by graft-derived neurons occurred in animals which received Saline-exposed NSC grafting (ACSF-exposed NSC, p<0.001 versus Saline-exposed NSC, p<0.05 versus PBS-exposed NSC; PBS, p<0.05 versus Saline-exposed NSC; n = 6 per group). These findings suggested an attenuated survival, integration, synaptogenesis and consequent therapeutic efficacy of harvesting media-exposed NSC.

### Effects on motor function recovery

**Figure 9:**
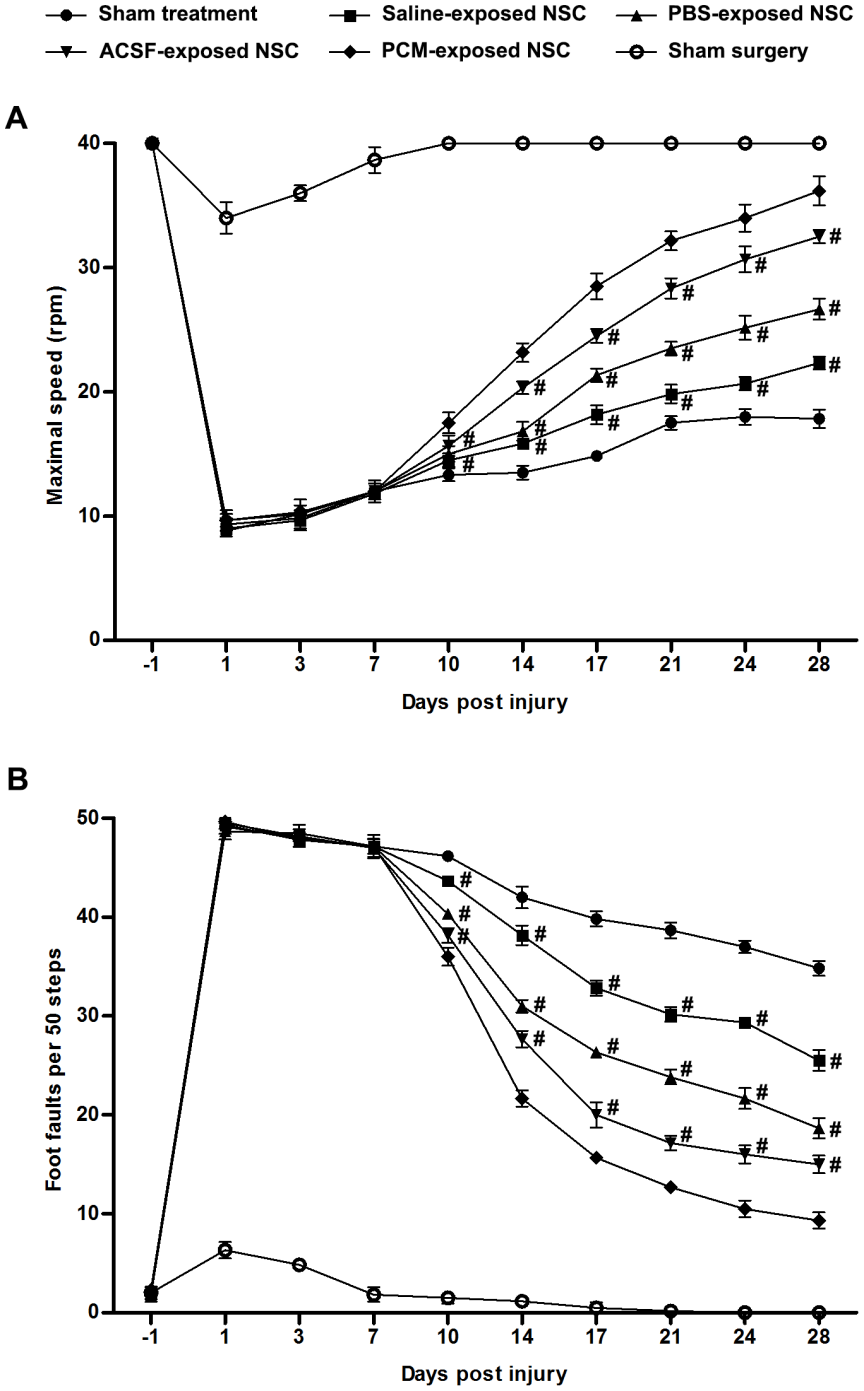
Effects on motor function recovery. (A) Quantitative analysis of the maximal speeds of mice in the rotarod test. (B) Quantitative analysis of foot faults per 50 steps of mice when walking across the beam. For (A) and (B), data are presented as mean ±SD; #, p<0.001 versus PCM-exposed NSC. n = 6 per group per time point. Transplantation was conducted 7 days post-TBI. Abbreviation: SD, standard deviation; TBI, traumatic brain injury.

To measure the impact of harvesting media exposure on the therapeutic efficacy of NSC, we conducted the rotarod test, which involves the examination of complex body movement and coordination. As shown in Fig. 9A, a notable functional impairment was observed in mice following TBI compared with those in the sham surgery group. In contrast with mice in the sham surgery group, the maximal speed at which mice were able to remain on the rotarod was significantly decreased in the TBI surgery group at day 1 post-TBI (Saline-exposed NSC, PBS-exposed NSC, ACSF-exposed NSC, PCM-exposed NSC and sham treatment, p<0.001 versus sham surgery; n = 6 per group), confirming the successful induction of TBI. Although mice with TBI displayed a gradual improvement in motor function over time (p<0.001, n = 6 per group per time point), no difference was detected before grafting (7 days post-TBI) between the treatment groups and the sham treatment group (Saline-exposed NSC, PBS-exposed NSC, ACSF-exposed NSC, and PCM-exposed NSC, p>0.05 versus sham treatment on 1, 3 and 7 days post-TBI; n = 6 per group per time point). From 3 days after the transplantation treatment (10 days post-TBI) to the end of observation (28 days post-TBI), mice in the treatment groups performed significantly better than those in the sham treatment group (Saline-exposed NSC, p<0.05 versus sham treatment at 10 days post-TBI; PBS-exposed NSC, ACSF-exposed NSC and PCM-exposed NSC, p<0.001 versus sham treatment at 10 days post-TBI; Saline-exposed NSC, PBS-exposed NSC, ACSF-exposed NSC and PCM-exposed NSC, p<0.001 versus sham treatment at 14, 17, 21, 24 and 28 days post-TBI; n = 6 per group per time point) and animals receiving the harvesting media injection showed no difference when compared with those in sham treatment group (Saline, PBS, ACSF or PCM, p>0.05 versus PCM at 10, 14, 17, 21, 24 and 28 days post-TBI; n = 4 per group; Fig. S3A). These data verified that NSC grafting was effective in the treatment of TBI and the benefits were not conferred by the harvesting media employed for transplantation. However, mice receiving grafting of harvesting media-exposed NSC performed significantly worse than those undergoing transplantation of PCM-exposed NSC (Saline-exposed NSC, PBS-exposed NSC and ACSF-exposed NSC, p<0.001 versus PCM-exposed NSC at 10, 14, 17, 21, 24 and 28 days post-TBI; n = 6 per group per time point; Fig. 9A), suggesting a reduced therapeutic efficacy of harvesting media-exposed NSCs.

In addition, the beam walk test was performed to evaluate the difference between the repair potential of NSCs exposed to the harvesting media or the PCM (Fig. 9B). At 1 day post-TBI, mice receiving TBI surgery displayed an obvious increase in foot faults when crossing the wooden beam (Saline-exposed NSC, PBS-exposed NSC, ACSF-exposed NSC, PCM-exposed NSC and sham treatment, p<0.001 versus sham surgery; n = 6 per group). Similar to the results of the rotarod test, the data from the beam walk test showed a time-dependent recovery of motor function of mice with TBI (p<0.001; n = 6 per group per time point). The beam walk test also showed that although NSC grafting contributed to functional recovery (Saline-exposed NSC, PBS-exposed NSC, ACSF-exposed NSC and PCM-exposed NSC, p<0.001 versus sham treatment on 10, 14, 17, 21, 24 and 28 day post-TBI; n = 6 per group per time point; Fig. 9B) and mere harvesting media injection was not capable of accelerating recovery (Saline, PBS, ACSF or PCM, p>0.05 versus PCM at 10, 14, 17, 21, 24 and 28 days post-TBI; n = 4 per group; Fig. S3B), the restoration of motor function in mice receiving harvesting media-exposed NSCs was attenuated compared to PCM-exposed NSCs (Saline-exposed NSC, PBS-exposed NSC and ACSF-exposed NSC, p<0.001 versus PCM-exposed NSC on 10, 14, 17, 21, 24 and 28 days post-TBI; n = 6 per group per time point; Fig. 9B).

Of note, among the harvesting media-exposed NSC treatment groups, TBI model mice that underwent ACSF-exposed NSC grafting performed best, PBS-exposed NSC-grafted animals came second and mice receiving Saline-exposed NSC grafting had the worst performance in both rotarod test (ACSF-exposed NSC, p<0.001 versus Saline-exposed NSC or PBS-exposed NSC on 14, 17, 21, 24 and 28 days post-TBI; PBS-exposed NSC, p<0.001 versus Saline-exposed NSC; n = 6 per group per time point; Fig. 9A) and beam walk test (ACSF-exposed NSC, p<0.001 versus Saline-exposed NSC or PBS-exposed NSC on 10, 14, 17, 21, 24 and 28 days post-TBI; PBS-exposed NSC, p<0.001 versus Saline-exposed NSC on 10, 14, 17, 21, 24 and 28 days post-TBI; n = 6 per group per time point; Fig. 9B). These data at the level of motor function recovery verified the advantage of the harvesting media containing glucose content or pH buffer system.

These results of morphological and behavioral assays that were consistent with and confirmed by each other demonstrated that the harvesting media indeed impaired the repair potential of NSCs after TBI.

## Discussion

Conventional harvesting media, including 0.9% saline, 0.01 M PBS and ACSF, are usually employed for the isolation, harvesting, sorting, testing and transplantation of NSCs [10]–[16], [30]. Among these solutions, PBS is most frequently selected as the vehicle for NSC suspension and transplantation [11]–[15], while ACSF is generally employed in electrophysiological assays [12], [16], [30]. Because of the physiological compatibility, these harvesting media are barely reported to have adverse effects on cells. Currently, almost no data exists regarding the effects of these solutions on NSCs and their post-mitotic progeny. Additionally, there is still no consensus on the optimal treatment conditions. However, due to the lack of trophic support provided by harvesting media and to variations in the treatment conditions, the characteristics and the repair potential of NSCs can vary. In this study, we explored the effects of different harvesting media and different treatment durations on the viability, proliferation, cell cycle progression, differentiation, apoptosis, necrosis and repair potential of NSCs. We sought to reduce the adverse effects and to maximize transplanted cell survival and functional recovery.

The viability and proliferation of NSCs are key factors in successful NSC transplantation [31], [32]. According to the criteria of the FDA, transplanted cells should maintain viability and proliferation levels above the permissible limit (70%) [23]. Our data indicate that following prolonged treatment, the viability and proliferation of NSCs gradually are deteriorated (Fig. 1C-E). The viability and proliferation of NSCs are influenced by multiple exogenous instructive factors, such as temperature, osmolarity, pH, and nutrients [33]–[39]. In the present study, we employed the harvesting media and the PCM with the same temperature (4°C) and osmotic pressure (280–310 mOsm). Therefore, the deterioration of the viability and proliferation of NSCs is not caused by the temperature or osmolarity of the harvesting media but likely associated with pH alteration (Fig. S4) and a lack of nutrients [5], [6], [9], [36]–[39]. The experimental harvesting media do not contain any growth factors and only provided short-term survival of NSCs. Deficiency or exhaustion of exogenous instructive factors or lack of pH buffer system may greatly inhibit the viability and proliferation of cells *in vitro*, while a greater number of NSCs survive in the media containing sufficient nutrients, powerful buffer system and essential growth factors [5], [38], [40]. Although we did not establish a relationship between the viability or proliferation of NSCs and the individual harvesting media in this study, our results suggest that NSCs cannot be maintained in the long term in the experimental harvesting media and indicate that appropriate harvesting media and appropriate treatment durations have benefits on cell survival and proliferation. However, it is striking that neurospheres can be maintained in the long term (more than 1 week) in the harvesting media (Fig. 2B,E). The mechanism underlying the differences in viability and proliferation and the tolerance to harvesting media exposure between dissociated NSCs and neurospheres remains to be identified.

There is abundant evidence for the coupling of proliferation and cell cycle progression to the nutrient environment and pH alteration [20], [41], [42]. We demonstrated that the exposure to the experimental group harvesting media (with relatively low nutrients or no pH buffer system) triggers p53-mediated cell cycle arrest and represses the expression of cyclin E1, eventually leading to the reduction of S-phase entry (Fig. 1D,E). However, our understanding of how the harvesting media exposure affects cell cycle progression is limited. Cell cycle arrest during starvation is often mediated by an accumulation of cyclin-dependent kinase inhibitors (CDKIs), such as p27 and p21 [25], [43]. However, p21, which acts downstream of p53, was not detected in this study, most likely due to its absence in embryonic stem cells as reported previously [24], [25]. Of note, NSCs were exposed to the harvesting media at 4°C where NSCs are isolated, harvested or transported. Although the internalization of EGF receptor (EGFR) which is considered as the main mitogen receptor is completely blocked at 4°C [44], [45], the EGF stimulation-induced phosphorylation and activation of EGFR and its effectors as well as their interaction are preserved and even enhanced [45]–[47]. These may act as the molecular basis of the EGF function on NSCs at 4°C, which conforms to the sustained proliferation of NSCs exposed to the PCM at 4°C (Fig. 1D). Collectively, our results suggest that NSC cell cycle progression is responsive to the nutrient content and pH of harvesting media.

Previous research indicates that growth factors have a significant survival function against cellular death [48]. The harvesting media employed in the current study do not contain any of the growth factors, thus deprivation of growth factors in the harvesting media may result in apoptosis and necrosis of NSCs. Our data indicate that harvesting media exposure-associated NSC death including apoptosis and necrosis occurs in a time-dependent manner (Fig. 1C, 3 and 4). The rate of apoptosis and necrosis in harvesting media exposure groups is Saline>PBS>ACSF. With prolonged treatment (longer than 8 h), the viable population of cells gradually shifts to the necrotic and apoptotic cell death population in the harvesting media exposure groups, and the progression is irreversible. Apoptosis is often initiated by either extrinsic or intrinsic signaling pathways [49], [50]. To further understand the molecular mechanism underlying NSC apoptosis, we examined the levels of apoptosis-related molecules. Our results indicate that following prolonged treatment duration, apoptotic pathways are activated. The expression of Fas-L and cleaved caspase 8, in NSCs are up-regulated, while activated caspase 9 in NSCs is not detectable (Fig. 4). Additionally, fluorescence labeling assays reveal the intact mitochondrial inner membrane (Fig. 5). According to our findings, it seems that the extrinsic pathway is involved in harvesting media exposure-associated NSC apoptosis. These findings coincide with previous studies showing that Fas is involved in apoptosis of embryonic cortical neuroblasts and in the development of fetal brain [51], [52].

Differentiation and proliferation are two sides of the same coin [5], [34], [40], [53], [54]. Although whether grafted NSCs mainly specialize into neuronal or glial lineage remains controversial, excessive astrogliosis may hamper axon outgrowth and reduce the function of NSCs [4], [6], [8], [28], [32], [55]. To determine whether harvesting media exposure could influence NSC differentiation, we identified post-mitotic cells that differentiated from NSCs. We found that PBS and ACSF do not affect cell differentiation significantly. However, exposure to Saline does not facilitate neuronal differentiation (Fig. 6C,D), suggesting that Saline exposure may attenuate *in vitro* neurogenesis probably due to the low pH. As expected, neurospheres unaffected by long-term harvesting media exposure-induced proliferation decline are also resist to neuronal differentiation deficiency after Saline exposure (Fig. S1). Our results indicate that *in vitro* differentiation of NSCs could be at least partly dependent on the type of harvesting media, although the signaling pathways mediating the harvesting media exposure-associated modulation of differentiation of NSCs remain to be determined.

Poor cell quality might lead to low viability and low levels of cell engraftment after transplantation [4]–[7], [9], [32], [56]. To evaluate whether harvesting media exposure affects morphological and functional recovery, treated EGFP-NSCs were grafted by *in situ* transplantation into the injured cortex. A considerably lower number of GFP^+^ cells migrate to the damaged site and integrated with the host tissue in the sections of low-scoring animals in various groups than in the sections of higher-scoring animals (Fig. 7–9), indicating that the functional test score parallels the number of GFP^+^ cells in the damaged site. Furthermore, the graft-derived cells in low-scoring animals bear obviously weaker competence of synaptogenesis than those in higher-scoring animals. Of note, the harvesting media-exposed NSC groups have fewer GFP^+^ cells as well as weaker synapse formation of graft-derived cells and low-scoring animals than the PCM-exposed NSC group. Therefore, our results indicate that harvesting media exposure determine, at least in part, the therapeutic efficacy of NSCs for TBI.

## Conclusions

In summary, harvesting media exposure inhibits the viability and proliferation of NSCs, reduces the S-phase entry, induces the apoptosis and necrosis of NSCs in a time dependent manner and attenuates NSC-mediated functional recovery. In order to maximize the repair potential of NSCs for TBI,we propose that cells should be exposed to those pH buffer system-containing and nutrient-enriched harvesting media and grafted within 6–8 h. Our findings suggest that an insight of the effects of harvesting media exposure on NSCs is critical for developing strategies to assure the successful long-term engraftment of NSCs.

## Supporting Information

Figure S1 **Effects of harvesting media exposure on the differentiation of NSCs in neurospheres.** (A) Immunofluorescence images of neurosphere cultures exposed to the harvesting media for 1 week, cultured in the differentiation medium for 14 days, stained for β-tubulin-III and counterstained by DAPI. (B) Quantitative analysis of β-tubulin-III-positive cells in neurosphere cultures exposed to the harvesting media for 1 week and differentiated for 14 days. Data are presented as mean ±SEM; ns, nonsignificant; n = 10 per group. Scale bar: (A)  = 100 µm. Abbreviation: DAPI, 4′,6′-diamidino-2-phenylindole; SEM, standard error of deviation.
(TIF)

Figure S2 **Damages of cortical tissue induced by TBI.** (A) Images of HE staining of TBI and sham surgery murine cerebral cortex. (B) Immunofluorescence images of TBI and sham surgery murine cerebral cortex stained for MAP2 and counterstained by DAPI. (C) TEM images of the murine cerebral cortex with TBI. (a) Arrow denotes an erythrocyte (hemorrhage; 0 d). (b) Arrow denotes a necrotic neuron (0 d). (c) Arrow denotes apoptotic neuron (0 d). (d) Arrow denotes microglia engulfing necrotic nervous tissue (5 d). Scale bar: (A)  = 200 µm; (B)  = 50 µm; (C)  = 2 µm. Abbreviation: DAPI, 4′,6′-diamidino-2-phenylindole; HE, hematoxylin and eosin; MAP2, microtubule-associated protein 2; NSC, neural stem cell; PCM, proliferation culture medium; TBI, traumatic brain injury; TEM, transmission electron microscopy.
(TIF)

Figure S3 **Motor function tests of TBI mice that received mere harvesting media injection.** (A) Quantitative analysis of the maximal speeds of mice in the rotarod test. (B) Quantitative analysis of foot faults per 50 steps of mice when crossing the beam. Injection was conducted 7 days post-TBI. For (A) and (B), data are presented as mean ±SD; n = 4 per group per time point. Abbreviation: SD, standard deviation; TBI, traumatic brain injury.
(TIF)

Figure S4 **pH changes of harvesting media and PCM as treatment went on.** Data are presented as mean ±SD; n = 5 per group per time point. Abbreviation: SD, standard deviation; PCM, proliferation culture media.
(TIF)
