# Supplementary figures and images for: The Effects of Harvesting Media on Biological Characteristics and Repair Potential of Neural Stem Cells after Traumatic Brain Injury
Source: PLoS One. 2014 Sep 23;9(9):e107865. doi: 10.1371/journal.pone.0107865 (PMC4172630; doi:10.1371/journal.pone.0107865)

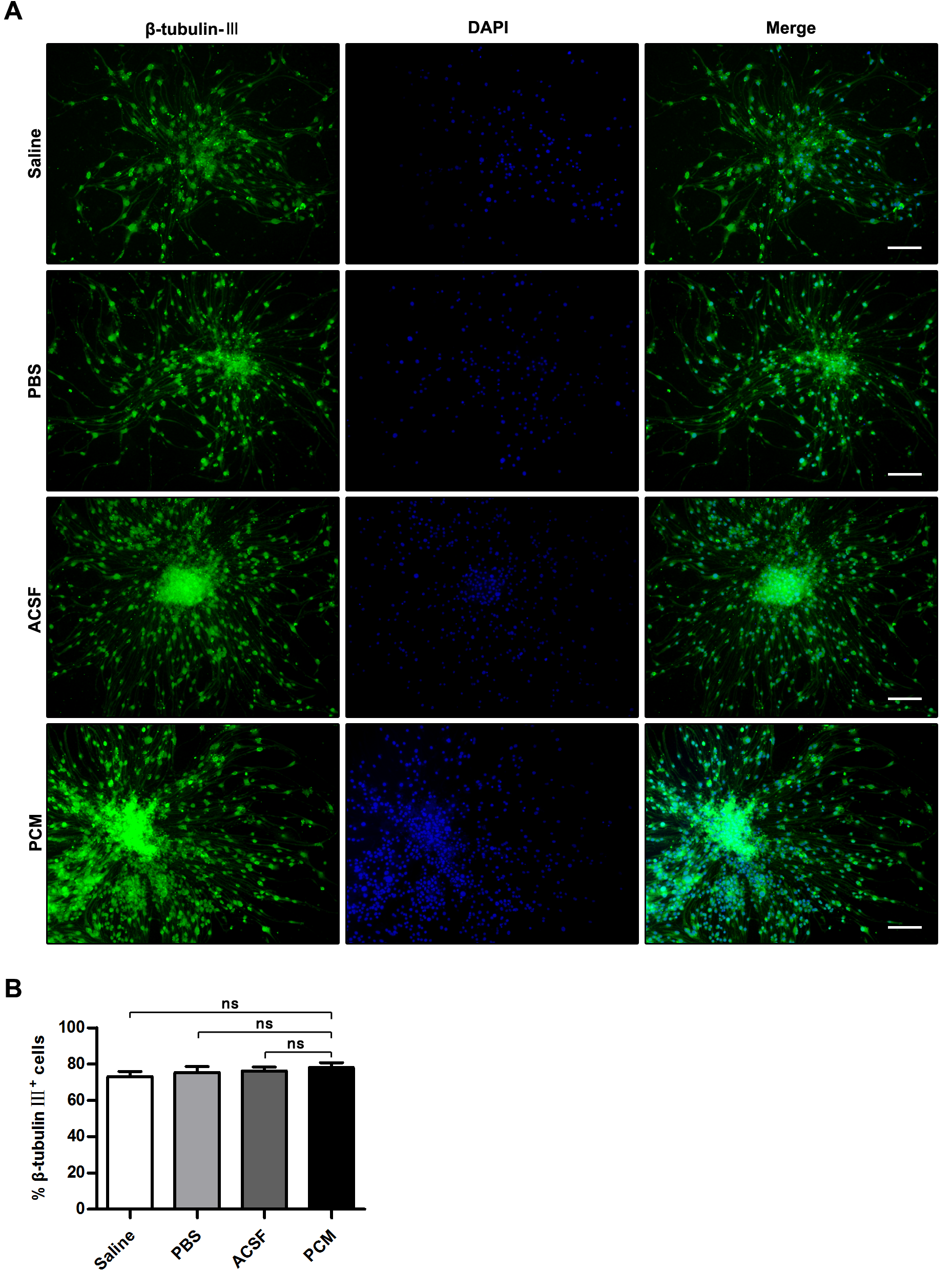

Supplement: Figure S1 — Effects of harvesting media exposure on the differentiation of NSCs in neurospheres. (A) Immunofluorescence images of neurosphere cultures exposed to the harvesting media for 1 week, cultured in the differentiation medium for 14 days, stained for β-tubulin-III and counterstained by DAPI. (B) Quantitative analysis of β-tubulin-III-positive cells in neurosphere cultures exposed to the harvesting media for 1 week and differentiated for 14 days. Data are presented as mean ±SEM; ns, nonsignificant; n = 10 per group. Scale bar: (A) = 100 µm. Abbreviation: DAPI, 4′,6′-diamidino-2-phenylindole; SEM, standard error of deviation. (TIF) [file pone.0107865.s001.tif]

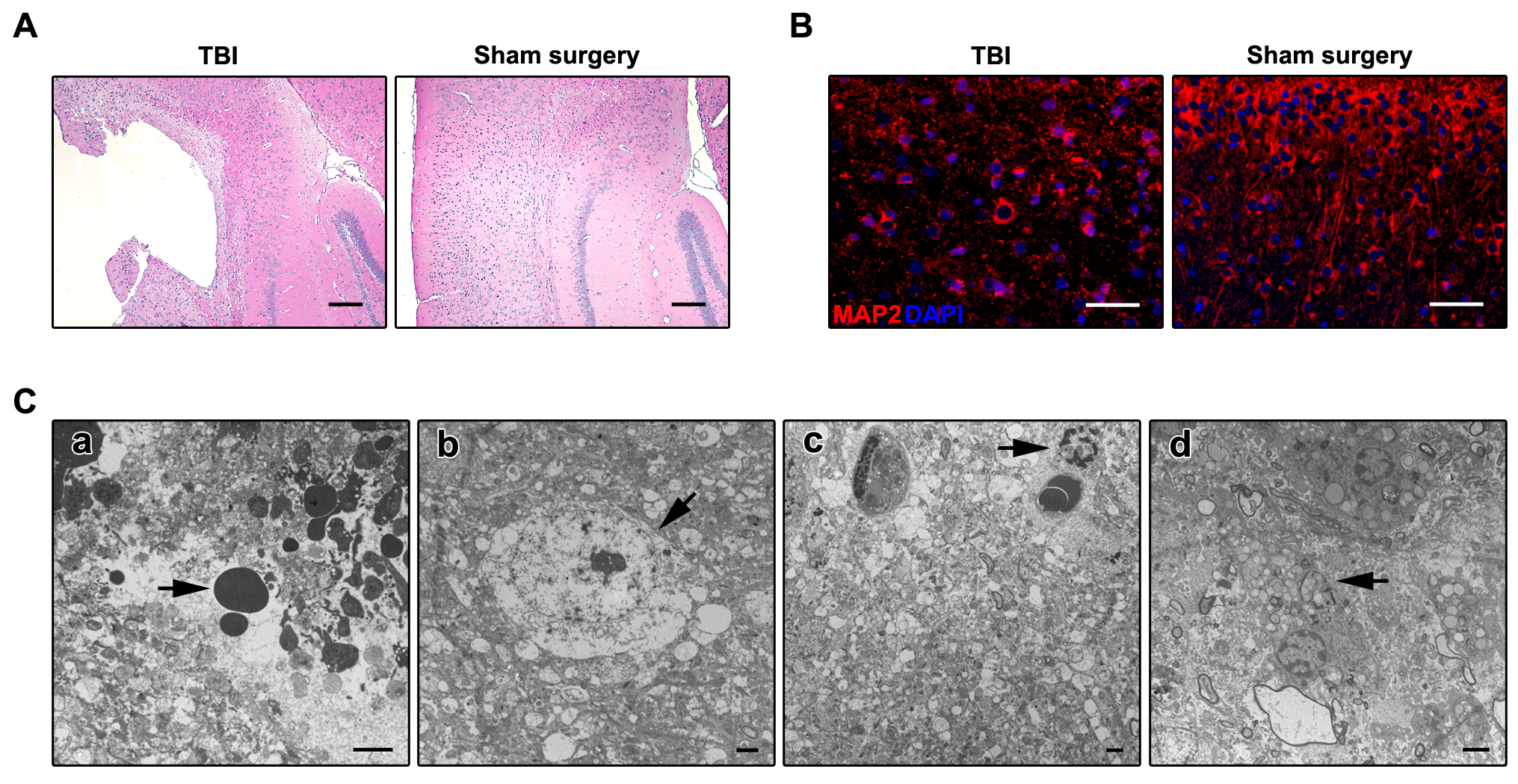

Supplement: Figure S2 — Damages of cortical tissue induced by TBI. (A) Images of HE staining of TBI and sham surgery murine cerebral cortex. (B) Immunofluorescence images of TBI and sham surgery murine cerebral cortex stained for MAP2 and counterstained by DAPI. (C) TEM images of the murine cerebral cortex with TBI. (a) Arrow denotes an erythrocyte (hemorrhage; 0 d). (b) Arrow denotes a necrotic neuron (0 d). (c) Arrow denotes apoptotic neuron (0 d). (d) Arrow denotes microglia engulfing necrotic nervous tissue (5 d). Scale bar: (A) = 200 µm; (B) = 50 µm; (C) = 2 µm. Abbreviation: DAPI, 4′,6′-diamidino-2-phenylindole; HE, hematoxylin and eosin; MAP2, microtubule-associated protein 2; NSC, neural stem cell; PCM, proliferation culture medium; TBI, traumatic brain injury; TEM, transmission electron microscopy. (TIF) [file pone.0107865.s002.tif]

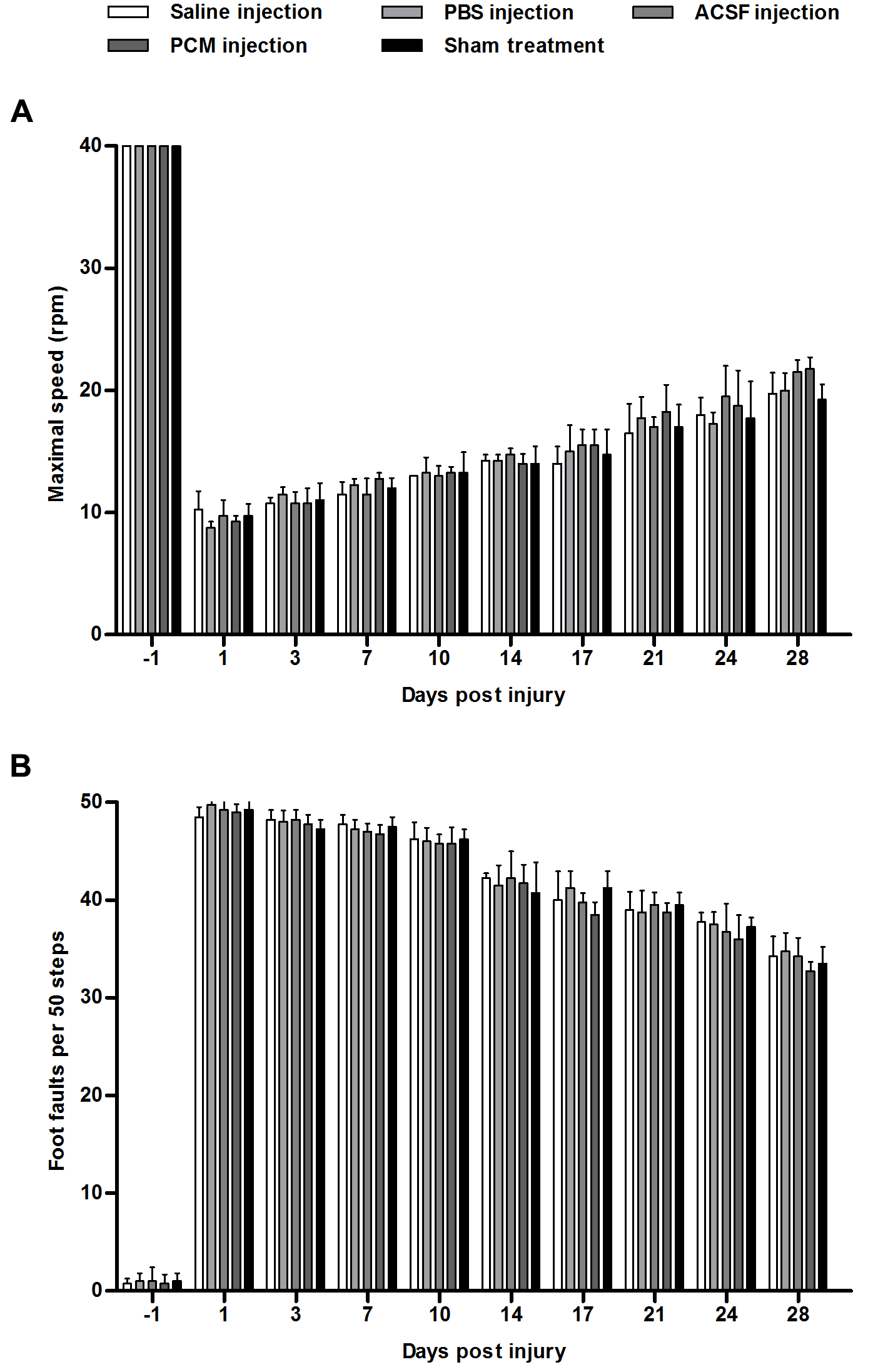

Supplement: Figure S3 — Motor function tests of TBI mice that received mere harvesting media injection. (A) Quantitative analysis of the maximal speeds of mice in the rotarod test. (B) Quantitative analysis of foot faults per 50 steps of mice when crossing the beam. Injection was conducted 7 days post-TBI. For (A) and (B), data are presented as mean ±SD; n = 4 per group per time point. Abbreviation: SD, standard deviation; TBI, traumatic brain injury. (TIF) [file pone.0107865.s003.tif]

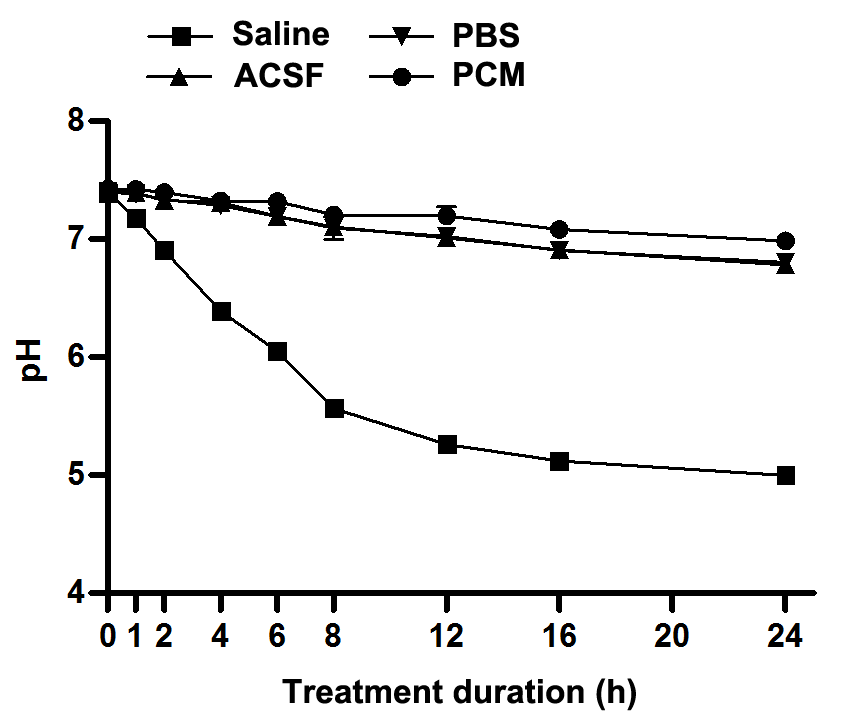

Supplement: Figure S4 — pH changes of harvesting media and PCM as treatment went on. Data are presented as mean ±SD; n = 5 per group per time point. Abbreviation: SD, standard deviation; PCM, proliferation culture media. (TIF) [file pone.0107865.s004.tif]
